# Composition and Dynamics of the Activated Sludge Microbiome during Seasonal Nitrification Failure

**DOI:** 10.1038/s41598-019-40872-4

**Published:** 2019-03-14

**Authors:** Juliet Johnston, Timothy LaPara, Sebastian Behrens

**Affiliations:** 1 University of Minnesota, Department of Civil, Environmental, and Geo-Engineering, 500 Pillsbury Drive S.E, Minneapolis, MN 55455-0116 USA; 2 University of Minnesota, BioTechnology Institute, 1479 Gortner Avenue, St. Paul, MN 55108-6106 USA

## Abstract

Wastewater treatment plants in temperate climate zones frequently undergo seasonal nitrification failure in the winter month yet maintain removal efficiency for other contaminants. We tested the hypothesis that nitrification failure can be correlated to shifts in the nitrifying microbial community. We monitored three parallel, full-scale sequencing batch reactors over the course of a year with respect to reactor performance, microbial community composition via *16S rRNA gene* amplicon sequencing, and functional gene abundance using qPCR. All reactors demonstrated similar changes to their core microbiome, and only subtle variations among seasonal and transient taxa. We observed a decrease in species richness during the winter, with a slow recovery of the activated sludge community during spring. Despite the change in nitrification performance, ammonia monooxygenase gene abundances remained constant throughout the year, as did the relative sequence abundance of *Nitrosomonadacae*. This suggests that nitrification failure at colder temperatures might result from different reaction kinetics of nitrifying taxa, or that other organisms with strong seasonal shifts in population abundance, e.g. an uncultured lineage of *Saprospiraceae*, affect plant performance in the winter. This research is a comprehensive analysis of the seasonal microbial community dynamics in triplicate full-scale sequencing batch reactors and ultimately strengthens our basic understanding of the microbial ecology of activated sludge communities by revealing seasonal succession patterns of individual taxa that correlate with nutrient removal efficiency.

## Introduction

Municipal wastewater treatment plants are designed to leverage the metabolic capabilities of microorganisms to remove excessive nutrients such as organic carbon, phosphorous, and ammonia from sewage. These diverse groups of microorganisms comprise the activated sludge microbiome which acts as the active biological component that remediates the influent wastewaters. One of the diverse microbial processes central to wastewater treatment is nitrification. Under aerobic conditions during wastewater treatment nitrifying microorganisms oxidize ammonia to nitrate. Under anaerobic conditions denitrifying bacteria can then reduce nitrate to gaseous forms of nitrogen (such as nitrous oxide and dinitrogen gas) ultimately lowering dissolved wastewater nitrogen concentrations. When untreated, elevated concentrations of ammonia, nitrite, and nitrate in rivers and streams contribute to eutrophication leading to hypoxic “dead zones” in receiving waters bodies, which constitute human and environmental health hazards. Among others these concerns have made managing the nitrogen cycle an important component of the grand challenges for environmental engineering in the 21^st^ Century^1^.

In temperate climate zones such as in many states in the Northern United States (e.g. Minnesota), wastewater treatment plants regularly experience a seasonal decrease in nitrification performance during the cold winter months. The loss in nitrification performance is often so prevalent that many local permits only require monitoring winter ammonia concentrations without any regulatory limits on effluent concentrations due to a lack of viable options for cost-effective removal of ammonia in the cold season. The discharge of elevated concentrations of ammonia (greater than 4 mg/L) with treated wastewater during the winter (water temperatures below 13 °C) is referred to as ‘seasonal nitrification failure.’ The State of Minnesota is working to incrementally reduce nitrogen loads to the Mississippi River by up to 40% by 2025 and even further in future years^2^. In order to be able to meet these discharge milestones, concerted efforts of wastewater treatment plant operators and microbiologists will be required to further our understanding of the impacts of temperate climate zone winter conditions on the composition and activity of the activated sludge microbiome at full-scale wastewater treatment plants.

The process of nitrification is commonly carried out by separate and taxonomically diverse groups of microorganisms^3^. First, ammonia-oxidizing bacteria and archaea oxidize ammonia to nitrite and then nitrite-oxidizing bacteria further oxidize nitrite to nitrate. Recently, also complete ammonia oxidizing bacteria (comammox) have been describe which are capable of oxidizing ammonia via nitrite to nitrate directly^4–7^. However, their role in domestic wastewater treatment requires further research.

Since heating wastewater is extremely costly, wastewater operators will typically react to seasonal nitrification failure by increasing the mixed-liquor suspended solids (MLSS), prolonging sludge aeration periods, and lengthening sludge retention times (SRT). These serve as attempts to increase nitrifying biomass and prolong reaction time but have not always been successful in preventing seasonal nitrification failure. Previous studies have often revealed conflicting results when analyzing the relationship of temperature to nitrifying biomass and plant nitrogen removal performance. Unfortunately, there is a lack of studies on full-scale sequencing batch reactors (SRBs) in temperate climate zones that also collected year-round seasonal data, so a lot of information about nitrification performance and nitrifying biomass abundance has been derived from laboratory experiments which are often not very representative of “real-world” scenarios. For example, in several laboratory studies using bench-scale membrane bioreactors and granular activated sludge systems on low temperature effects on the abundance of nitrifying biomass and reactor nitrification performance have been observed^8–11^. This might indicate that systems operating with prolonged retention times are less affected by temperature fluctuations^9,12^. However, other laboratory studies using reactors operated similarly to full-scale sequencing batch reactors (SRBs) have reported a direct correlation between loss of nitrifying biomass and decreasing nitrogen removal efficiency^13–18^. Still other laboratory studies have observed a decrease of ammonia removal efficiency at lower temperatures with no decrease in the abundance of ammonia-oxidizing bacteria, but notable fluctuations in the abundance of nitrite-oxidizing bacteria^19–21^.

Only a few studies of microbial community dynamics in full-scale activated sludge systems in temperate climate zones have been performed using high-throughput microbiome analyses to compare community dynamics to ammonia removal efficiency and seasonal temperature fluctuations^6,16,21–27^. These studies have shown that the activated sludge microbial community is shaped to some extend by both deterministic and neutral factors^21,26–30^. Shifts in wastewater treatment plant nitrification performance have been also previously studied by quantifying ammonia monooxygenase (*amoA*) gene copy numbers using qPCR to monitor the seasonal dynamics of ammonia- oxidizing bacteria (AOB)^13,15^. However, nearly all current full-scale wastewater treatment plant studies have investigated continuously stirred tank reactor configurations (CSTRs) which operate at steady-state, while information on the seasonal community dynamics in sequencing batch reactors that operate under dynamic feast-famine conditions is scarce.

Here we provide a detailed analysis of the composition and seasonal dynamics of the activated sludge microbial community in three parallel SBR of a wastewater treatment plant that almost completely loses its nitrification performance each winter. The WWTP at Brainerd Minnesota is a great sampling location to address the microbial ecology of seasonal nitrification failure because of its geographic location (Coordinates: 46°21′29″N 94°12′03″W), its continental climate with vast seasonal temperature differences (average high 26.9 °C in July and average low −19.8 °C in January), and because of the fact that the plant operates three parallel SBRs at any given time which allowed for replicate sampling of full-scale reactors at high temporal resolution (weekly) for over a year.

The objective of this study was to identify and quantify the abundance dynamics of core and seasonal microbial taxa in the activated sludge of three parallel, full-scale sequencing batch reactor. Shifts in abundance of microbial taxa were correlated to observed conditions of ‘nitrification failure’, defined as decrease in nitrification performance during winter months. We link relative sequencing abundance from high-throughput sequencing to absolute gene abundances determined by quantitative polymerase chain reaction (qPCR). Emphasis was on ‘known’ nitrifying microbial groups including ammonia-oxidizing bacteria, archaea and nitrite-oxidizing bacteria. We identify a ‘core’ community of microbial taxa that were present year-round in all three reactors, seasonal taxa that were associated with only one or multiple seasons but that were non-detectable during at least one of the four seasons, and transient taxa that were present in any of the three reactors for shorter time period but were never consistently found. We define ‘abundant’ microorganisms in each of these groups and correlate their occurrence and seasonal dynamics with wastewater temperature and effluent ammonia concentrations throughout the year. This work identifies important activated sludge microorganisms that affect the nitrification performance of SRBs. Overall, this work contributes to a better understanding of the microbial community composition and dynamics of activated sludge systems in SBRs in temperate climate zones that are experience strong seasonal temperature fluctuations.

## Materials and Methods

### Plant and sampling

The Brainerd Wastewater Treatment Facility (WWTF) in Minnesota is a Class A facility designed to treat domestic wastewater at an average wet weather flow of 22,700 m^3^/day with a carbonaceous biological oxygen demand (CBOD) influent concentration of 240 mg/L and a TSS concentration of 240 mg/L. The average observed influent ammonia concentration is 35 mg/L throughout the year, while nitrate and nitrite were not detected. The average influent flow during the study period ranged from 6,965 ± 303 m^3^/day in winter 2015 to 9,577 ± 2640 m^3^/day in summer 2016. The Facility has a continuous discharge to the Mississippi River.

The influent domestic wastewater is pumped into one of three parallel sequencing batch reactors (SBRs) at a time. Once a reactor has been filled aeration starts and the influent wastewater is directed into the next reactor. This process is repeated about every two hours allowing for nearly identical treatment conditions in three completely independent activated sludge reactor systems. Aeration is pulled from ambient air without prior heating. Each reactor cycle lasts approximately six hours. During each six-hour cycle reactors operational conditions switch from static filling, mixed filling, aeration, settling, decanting and waste sludge removal. The reactors are located underground and completely covered which reduces seasonal temperature variations and limits photosynthetic growth. In summer 2016, during sampling weeks 53 to 55, heavy rainfalls increased influent flow rates from 7000 m^3^/day to 9500 m^3^/day resulting in slightly lower sludge retention times of 4.5 days instead of 8 days during the rest of the year.

Activated sludge samples were collected for 55 consecutive weeks (July 13, 2015 to July 31, 2016) from each of the three parallel reactors in operation. The samples were always taken about one hour into the aerobic reaction cycle following reactor filling. Each reactor is connected to a sink for easy sampling. Prior to sample collection pipes were flushed for about 5 min each time. Collected wastewater samples were immediately frozen at −20 °C until further processing. At week 18 the impellor of SBR 1 broke. The activated sludge of SBR 1, as well as some sludge of reactor SBR 2 and 3 were transferred into SBR 4 at this time to continue operations with three reactors. The initial startup of SBR 4 marked the only time during our year-long sampling campaign at which reactor contents were mixed and therefore not operated completely independent of each other. SBR 2 and 3 only donated activated sludge to SRB 4 during its initial startup. Otherwise SBR 2 and 3 were completely independent during the entire year-long experiment. Since at any given time of the year-long sampling campaign the wastewater treatment plant operated with three parallel SBRs we decided to combine the datasets of week 1 to 17 of SBR 1 with weeks 19 to 55 of SBR 4. Because of the failure of SBR 1 during week 18 we removed the sample from the dataset. For consistency in the following data analysis we will only refer to the three parallel reactors as SBR 1, 2, and 3 with SBR 1 being the reactor that was restarted at week 18.

The plants performance data was made available to us by the wastewater facility in Brainerd, MN. Total suspended solids (TSS), pH, water temperature, biological oxygen demand (BOD), sludge volume index (SVI), mixed liquor suspended solids (MLSS), sludge blanket, ammonia and phosphorus concentrations were determined according to standard EPA methods. A summary of the plant chemical and operational parameters is shown in Table S1 in the Supplementary Information. This includes daily temperatures, influent flow rates, and pH. Several parameters such as mixed-liquor suspended solids, BOD, and phosphorous are recorded 3 times per week. In this case we report weekly averages for that specific data in Table S1. The treatment plant only measured ammonia concentrations every 2–4 weeks based on their permit requirements. Due to the infrequency of ammonia testing, additional samples were collected before nitrification failure began.

Starting January 2016 (week 26) effluent wastewater samples from the reactors were collected to quantify ammonia, nitrite, and nitrate concentrations using continuous segmented flow analysis to complement the chemical data routinely monitored by the plant. Effluent samples were taken from an equalization basin that receives the supernatants of all SBRs and immediately frozen at −20 °C until analysis. This means that the collected data on effluent ammonia, nitrite, and nitrate concentrations represent average values for the three operational SRBs.

### Continuous Segmented Flow Analysis

Quantification of ammonia, nitrate, and nitrate concentrations in effluent wastewater were performed on a SEAL AutoAnalyzer 3 HR continuous segmented flow analyzer (Seal Analytical Inc; Mequon WI) according to standard multi-test methods as to manufacturer’s instructions. In brief, effluent wastewater was diluted 1:4 with deionized water and centrifuged for 5 min at 13,000 g to collect the supernatant. Ammonia concentrations were quantified using method No. G-102-93 (with salicylate chemistry), detection range 0.25 to 25 mg/L as nitrogen. Quantification of nitrate and nitrite concentrations were performed using method No. G-109-94 with hydrazine sulfate for NO_x_ measurements and without hydrazine sulfate for NO_2_^−^ quantification, detection range 0.3 to 11 mg/L as nitrogen. NO_3_^−^ concentrations were calculated by subtracting NO_2_^−^ concentrations from NO_x_ concentrations.

### 16S rRNA gene amplicon sequencing

DNA was extracted from the activated sludge samples using the *FastDNA*^*TM*^ *SPIN Kit for Soil* (MP Biomedicals; Santa Ana; CA) according to the manufacturer’s instruction with the following modifications. Sludge samples were thawed on ice and thoroughly mixed before 50 µL were mixed with 450 µL of 5% (v/v) sodium dodecyl sulfate lysis buffer (120 mM sodium phosphate (pH 8.0) and 5% sodium dodecyl sulfate lysis buffer). Cell lysis was performed by three consecutive freeze-thaw cycle at −20 °C, thawing samples on ice in between freezing. Following cell lysis samples were incubated for 90 min at 70 °C in a water bath. The following steps were carried out as to the *FastDNA*^*TM*^ *SPIN Kit* protocol. The DNA extracts were stored at −20 °C until further use.

DNA extracts were provided to the University of Minnesota Genomics Center (UMGC) for 16S rRNA gene amplicon sequencing, beginning from DNA quality assessment and quantification, barcoded amplification, PCR product purification and library preparation. Protocols for each step followed UMGC-developed methods, which have been published previously by Gohl *et al*.^31^. Sequencing primers were those also used for the Earth Microbiome Project covering variable regions V1 through V3 on the 16S RNA gene. The choice of sequencing primers follows the recommendation of Albertsen *et al*.^32^ for the phylogenetic analysis of activated sludge communities^32^. Controls using PCR water for DNA extraction and PCR amplification were included to verify reagent purity and identify potential sample contamination during library construction. Sequencing of all samples and controls was performed on an Illumina MiSeq sequencing system (Illumina, San Diego, CA, USA) using the 2 × 300 bp MiSeq Reagent Kit v3 (600 cycle) (Illumina, San Diego, CA, USA). The MiSeq Reporter Software v2.5.1.3 (Illumina, San Diego, CA, USA) was used for signal processing, de-multiplexing and trimming of adapter sequences.

### Microbial diversity analysis

Sequence analysis was performed using the Gopher-Pipeline of the University of Minnesota Supercomputing Institute^33^. This pipeline utilizes PandaSeq to stitch primers together, QIIME, ChimeraSlayer’s usearch61 method for chimera detection within QIIME. Default settings were used for quality control, primer trimming, filtering, chimera and host detection. Subsampling and rarifying samples were disabled in the pipeline. Operational taxonomic unit (OTU) picking was done outside of the Gopher-Pipeline using QIIME and the SILVA rRNA database (release 128)^34,35^. OTUs were assigned at the 97% cutoff level. Only two samples, SBR 2, week 8 and SBR 3 week 2 did not amplify and were disregarded from the dataset. DNA extraction and PCR control samples resulted in >5000 low quality sequence reads. Plotting the number of sequence reads against unique OTUs (data not shown) demonstrated that the diversity of the all controls was significantly different from all activated sludge samples.

### Statistical analysis

The statistical analysis of the obtained sequence data was performed using R version 3.4.2^36^. Ordination was performed using the *vegan software* package in R while regression analysis, ANOVA and ANCOVA functions, as well as comparative t-tests were done using the *alr4* software package in R^37,38^. ANOVA was used for linear regression analysis which was performed to determine relationships over time, or temperature changes. If the null hypothesis was zero, and no linear relationship was discernable, a comparative t-test was performed to compare the statistical means for the two groups. The 95% confidence intervals for statistical mean values were calculated using R and are provided when comparing linear regressions which could disprove the null hypothesis. ANCOVA was used in specific cases to determine if multiple factors were impacted by a single response such as total biomass and temperatures effects on the ammonia oxidizing community. Constrained Correspondence Analysis (CCA) was performed using the vegan package in R, which utilizes the Chi-squared distances. The analysis was performed twice to separate variables which can be controlled by plant operators, and all environmental parameters which are uncontrollable. Temperature was used in both analyzes because the parameter can potentially be controlled by plant operators, while it’s also an intrinsic characteristic of the influent wastewater. All statistical values are provided in the Supplementary Information.

### Quantitative Polymerase Chain Reaction

Quantification of 16S rRNA and functional marker genes was performed on a 7900HT Fast Real-Time PCR System with a 384-well block module (Applied Biosystems Inc, Foster City, CA). Reactions were performed in 25 µL volumes. Each reaction mix contained 10 µL of PCR Grade Water (Ambion Inc; Foster City, CA), 12.5 µL of 2X SsoFast^TM^ EvaGreen® Supermix (Bio-Rad Laboratories; Hercules, CA), 1.25 µL of 10 mg/L bovine serum albumin solution (Millipore Sigma; St. Louis, MO), 0.5 µL of forward primer (0.5 µM), 0.25 µL of reverse primer (0.5 µM), and 0.5 µL of template DNA.

Gene quantification by qPCR targeted the *16S rRNA* gene as proxy for total Bacteria, and the ammonia monooxygenase genes (*amoA)* of Bacteria and Archaea to estimate the abundance of nitrifier populations. Functional marker gene quantification to estimate population abundance of denitrifiers targeted the nitrite reductase genes *nirK* and *nirS* as well as the nitrous oxide reductase gene (*nosZ clade 1)*. Primer sequences and references are provided in Table S2 in the Supplementary Information. PCR cycler temperature programs for the *16S rRNA* and *amoA* gene of Bacteria comprised 40 cycles of 15 sec at 95 °C and 1 min at 60 °C. Both assays for the *amoA* gene of Archaea and the *nosZ* gene had 40 cycles of 15 sec at 95 °C followed by 1 min at 54 °C. The *nirK* and *nirS* gene qPCR assay temperature programs comprised 40 cycles of 15 sec at 98 °C, 54 °C for 30 s, and an elongation step of 30 sec at 72 °C. As standards, dilution series of linear DNA fragments (g-Blocks, IDT DNA Technologies) of the respective target genes were used. All samples, standards, and negative controls were analyzed in triplicates and amplicon specificity was confirmed by melt curve analyzes. Amplification efficiencies of the different assays ranged from 81% to 103% (average of 88%). The R^2^ values of the standard curves ranged from 0.983 to 0.999 (average of 0.9926).

## Results

### **Seasonal variations in plant nitrification performance**

**Figure 1:**
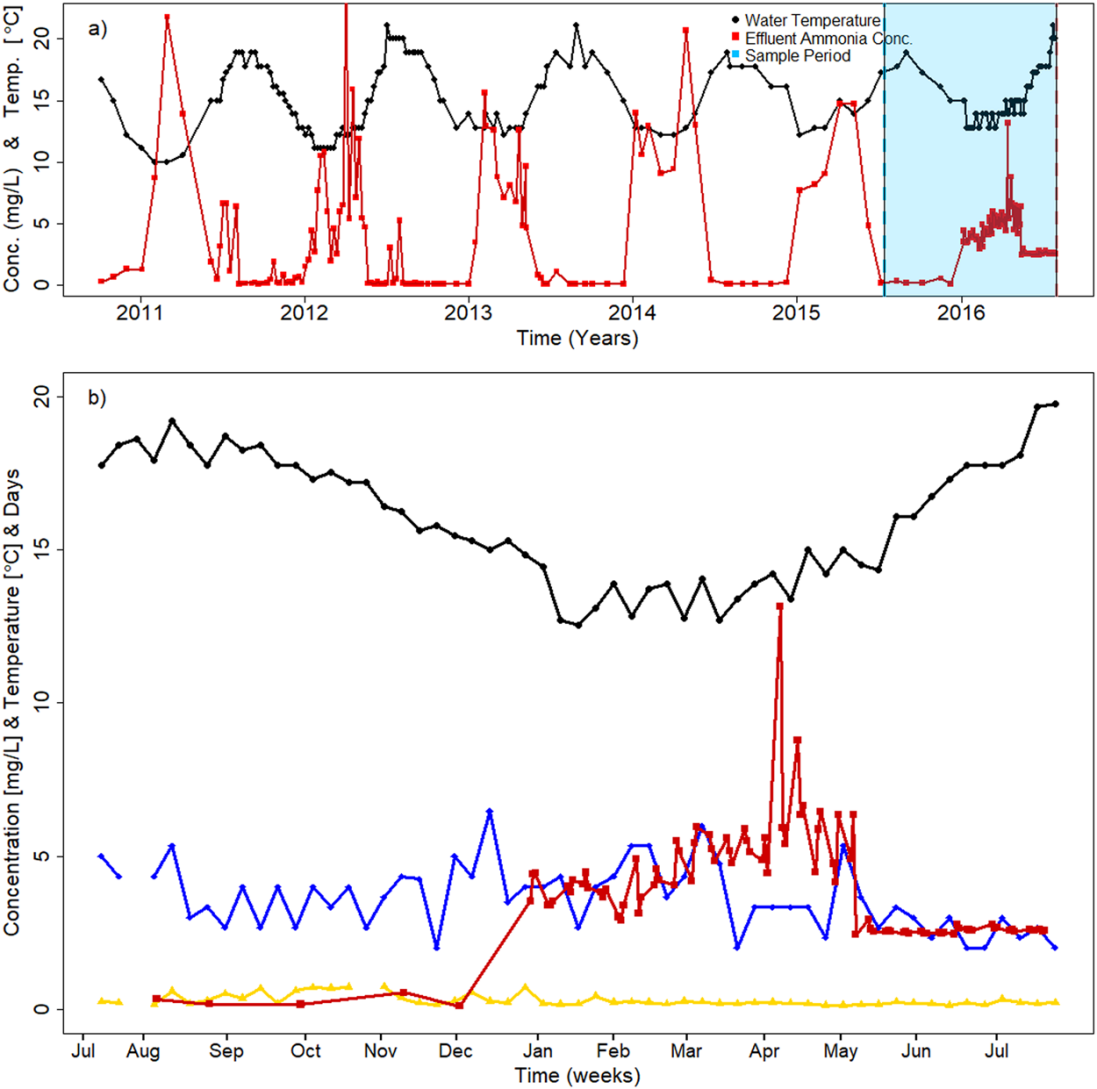
Brainerd wastewater treatment plant performance parameters from 2011 to 2016. (**a**) Influent water temperature (black circles) and effluent ammonia concentration (red squares) for all years since plant start-up in 2011. The sampling year summer 2015 to summer 2016 is highlighted in light blue. (**b**) Plant performance parameters for the sampling period from July 2015 to July 2016. Influent water temperature (black circles), effluent ammonia concentration (red squares), effluent biological oxygen demand (BOD) (blue diamonds), and effluent phosphate concentrations (yellow triangles). Effluent ammonia concentrations were inversely correlated to temperature.

The SBRs of the wastewater treatment facility in Brainerd Minnesota were seeded with activated sludge in September 2010. All three parallel reactors went ‘online’ beginning of 2011. Since start-up of the plant, the influent wastewater temperature fluctuates seasonally from as low as 10 °C in the winter to up to 20 °C during the summer month. Influent wastewater temperature fluctuations are inversely correlated to effluent wastewater ammonia concentrations. With decreasing wastewater temperatures, nitrification performance declines resulting in elevated effluent water ammonia concentrations (Fig. 1). Figure 1 shows that ammonia concentrations increase at influent wastewater temperatures around 13 °C and continue to rise as long as colder temperatures prevail in the winter month. The most significant increase in effluent ammonia concentration during our sampling period occurred between December 2015 (average 0.43 ± 0.42 mg/L) and January 2016 (average 4.48 ± 4.27 mg/L) (p < 0.005). When water temperatures rise in the Spring, ammonia effluent concentrations begin to decrease again. However, recovery of complete ammonia removal takes several months, often until May or June, even after wastewater temperatures have risen again above 13 °C. While permit requirements require plant operators to monitor ammonia concentrations in the wastewater effluent periodically, influent wastewater ammonia concentrations are only measured sporadically. Influent wastewater ammonia concentration at the sampled wastewater treatment plant were average for standard domestic wastewater and only slightly fluctuated around 35 mg/L^39^ throughout the sampling year.

Based on the annual wastewater influent temperatures and effluent ammonia concentrations for the sampling period July 2015 to July 2016, we defined five seasons as follows: Summer (July 13^th^ to September 22^nd^, 2015), Fall (September 23^rd^ to December 20^th^, 2015), Winter (December 21^st^, 2015, to March 18^th^, 2016), and Spring (March 19^th^ to June 19^th^, 2016). Sampling continued into Summer 2016 until July 31^st^, 2016.

Over the course of the 55-weeks of sampling, the influent wastewater temperature range fluctuated by 9 °C with a high of 21 °C in summer 2015 and a low of 12 °C during the winter month. The rate of temperature decrease/increase between the cooling period (Fall and Winter month) and the warming period (Spring and Summer month) for the sampling year was ±0.043 °C/day (Fig. S1).

Effluent ammonia concentration was the only routinely monitored wastewater parameter that showed significant seasonal fluctuation (p < 0.005), ranging from as low as 0.1 mg/L (method detection limit) in the Fall to 8.2 mg/L in Spring. Other parameters such as Biological Oxygen Demand (BOD_5_), phosphate concentrations (P), and Total Suspended Solids (TSS) did not vary significantly with season (p < 0.74, 0.41, and 0.20, respectively, as shown in Fig. S1). A detailed list of average plant operational parameters for each season is provided in Table S1 in the Supplementary Information.

### **Seasonal community dynamics**

#### *Outcome sequencing data – general overview*

The seasonal shifts in community composition in the three SBRs were analyzed using barcoded 16S rRNA gene amplicon sequencing. Sequencing resulted in 3.95 × 10^7^ total reads. 92.8% of the raw reads passed the initial quality filtering. Of the remaining 3.67 × 10^7^ reads 76.9% of the bases had a Q-score greater than 30. Chimera detection removed additional sequences so that in the end in 7.58 × 10^6^ non-chimeric, quality-filtered reads (on average 56,000 reads per sample) were subjected to OTU clustering at the 97% cutoff level. OTU clustering resulted in 1984 unique ‘species-level’ OTU’s for the total sequence data set. For analysis and better comparison of samples, we assumed that the read abundance, meaning the number of reads in each OTU, is corresponding to the actual abundance of the respective 16S rRNA phylotype in each activated sludge sample. However, it is important to keep in mind that the read abundance in DNA amplicon sequencing data sets is affected by DNA extraction efficiency, primer specificity, and the copy number of ribosomal RNA operons per genome and does not reflect ‘real’ natural abundance.

#### *Alpha diversity and reactor synchron*y

We calculated the alpha diversity indices, Simpson, Shannon, and Chao1 in order to compare the local species diversity of the activated sludge communities for each of the three SBRs over the complete sampling period of 55 weeks^40–42^. All three alpha diversity indices did not vary significantly with time considering the entire sampling period (Fig. S2a–c). The differences in the Simpson and Shannon indices between reactors were mostly not significant (p > 0.05) (Table S3). Significant differences in alpha diversity between reactors were only observed for reactors 1 and 2 as well as 2 and 4 (Table S3). This was most likely a consequence of the shutdown of reactor 1 at week 18 and transfer of activated sludge from reactors 1, 2 and 3 as inoculum to start up reactor 4 (in the following we refer to reactor 4 as reactor 1, see further explanations in the methods section). Reactors 2 and 3 were operated independently over the whole sampling period, however no significant differences in alpha diversity indices were observed over the entire year of sampling comparing these two reactors. The average alpha diversity indices for the three operational reactors at any given time during the sampling year were 0.97 ± 0.01, 4.53 ± 0.18, and 625 ± 87 for the Simpson, Shannon, and Chao1 indices, respectively.

While alpha diversity did not significantly vary between reactors, we observed significant differences when comparing the Simpson and Shannon indices for the different seasons. Generally, diversity was lower in the winter and higher in the summer. Including fall and spring the microbial community diversity in the reactors varied significantly each season (p > 0.05) (Table S4). Exceptions were the Winter/Spring transition for Shannon and Simpson indices at class-level OTU clustering and the Summer/Fall transition for the Simpson index at genus-level OTU clustering (Table S4). No seasonally significant differences were observed for the Chao1 index, which fluctuated week-to-week but was consistent among the reactors (Fig. S2c).

#### *Definitions: core & seasonal community (one vs multiple seasons)*

In order to study the annual shifts in the activated sludge microbial community composition and identify microbial taxa associated with seasonal shifts in wastewater temperature and effluent ammonia concentrations we group the total 1984 species-level OTUs into four categories: A ‘core’ community of microbial taxa that were present year-round in all three reactors, ‘single season’ taxa that were found during only one specific season, ‘multiple season’ taxa that were found during more than one season but that were absent during at least one of the four seasons, and ‘transient’ taxa that were present in any of the three reactors for shorter time periods (only a few weeks) but were never consistently found during an entire season or for any longer consecutive period of time.

**Figure 2:**
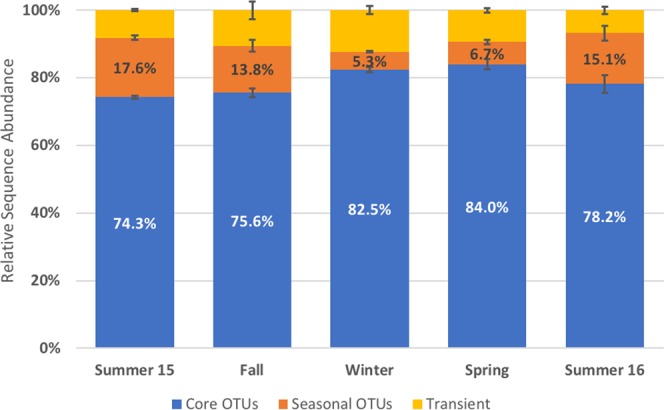
Average abundance of core, seasonal, and transient operational taxonomic units (OTUs). Core OTUs (blue) were present year-round in all three reactors. Seasonal OTUs (orange) were found during only one specific season or multiple seasons but were absent during at least one of the four seasons. Transient OTUs (yellow) were present in any of the three reactors for shorter time periods (weeks) but were never consistently found during an entire season or for any longer consecutive period of time. The relative sequence abundance of seasonal OTUs decreases during the winter and increases in the summer month.

The core community comprised 114 OTUs occurring in all three operational reactors at any time during the sampling year (Fig. S11). This ‘core’ community constituted the largest fraction of the entire activated sludge community with a relative sequence abundance of 74.3% and 78.2% during the two summers 2015/16, 82.5% in winter of 2015, and 84.0% in Spring 2016 (Fig. 2). Combining the two categories of seasonally occurring OTUs, they accounted for 15.1% and 17.6% of the activated sludge community in summers 2015/16, and only 5.3% and 6.7% in winter and spring. We identified more seasonal OTUs in the summers 2015/16 and fall (96/105 and 75 OTUs, respectively) than in the winter and spring (39 and 55 OTUs) (Fig. S11). Transiently occurring OTUs ranged in relative sequence abundance from 6.7% to 12.3%, with relatively higher abundances in the winter than in the summer. While transiently detected OTUs made up only a relatively small fraction of the entire community in each season this category had the highest OTU richness with numbers between 334–346 in the summer and up to 372 OTUs during the winter (Fig. S3 in the Supplementary Information).

#### OTUs that change with plant nitrification performance and temp

**Figure 3:**
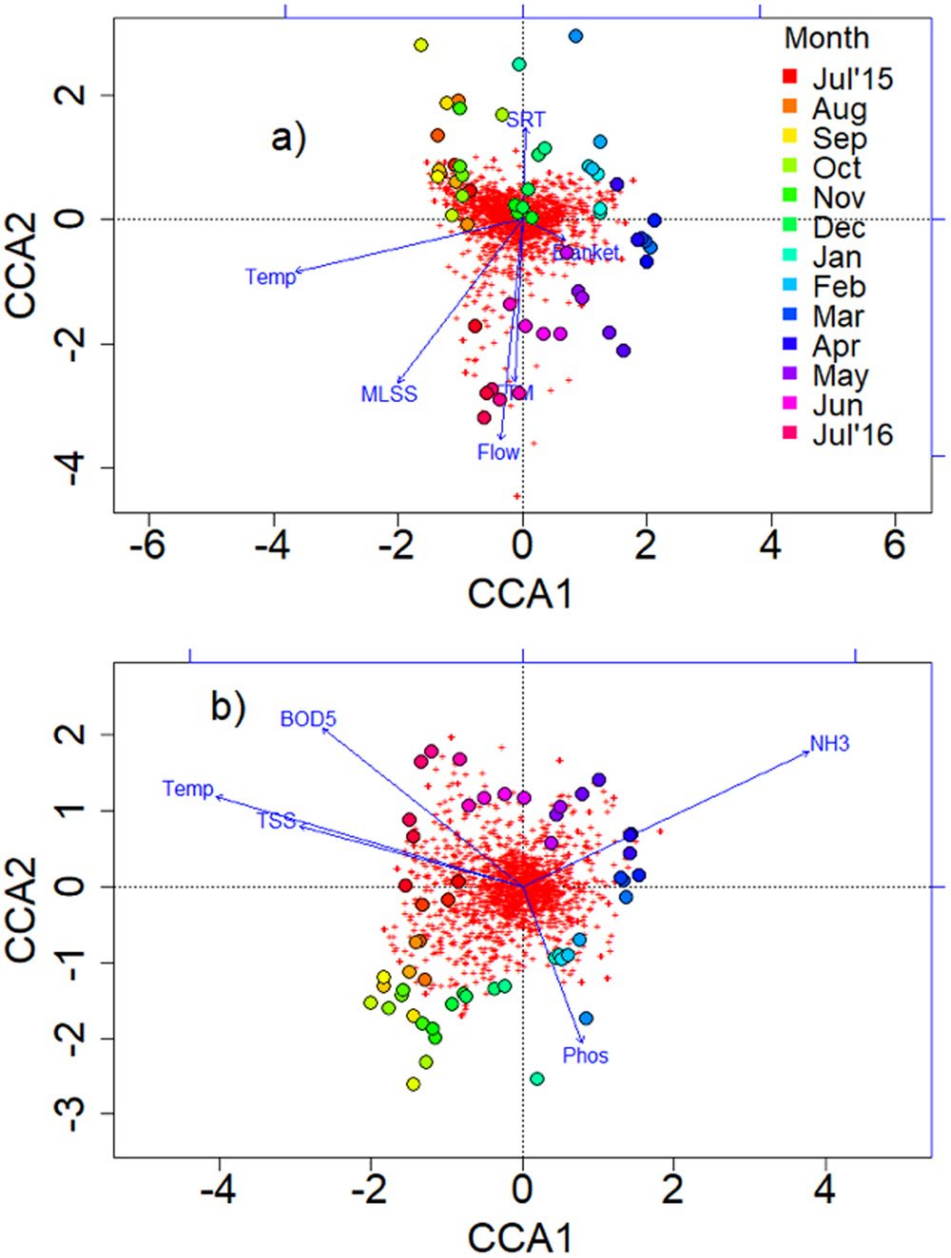
Constrained correspondence analysis (CCA) with operational and performance parameters with the activated sludge microbial community composition at the Brainerd wastewater treatment plant for July 2015 to July 2016. All classified OTUs in the complete sequence dataset are shown as red dots. The larger rainbow-colored dots represent the 55 weekly average microbial communities for the three sampled sequencing batch reactors. Parameter variable are shown as blue arrows and include temperature (Temp), mixed liquor suspended-solids (MLSS), influent flow rate as m^3^/day (Flow), food-to-microbes ratio (FTM), sludge blanket levels (Blanket), sludge retention time (SRT), effluent ammonia (NH3), biological oxygen demand (BOD5), phosphorous concentration (Phos), and total suspended solids (TSS). (**a**) The CCA including the five recorded operational parameters MLSS, Flow, FTM, Blanket, and SRT. (**b**) CCA including the four plant performance parameters NH3, BOD5, Phos, and TSS. Temperature as potentially controllable parameter was included in both plots to emphasize its strong effect on community composition and structure.

Canonical-correlation analysis (CCA) reveal that wastewater temperature was the most dominant variable affecting microbial community composition and shift throughout the year. Effluent ammonia concentration was inversely correlated to changes in wastewater temperature (Fig. 3).

**Figure 4:**
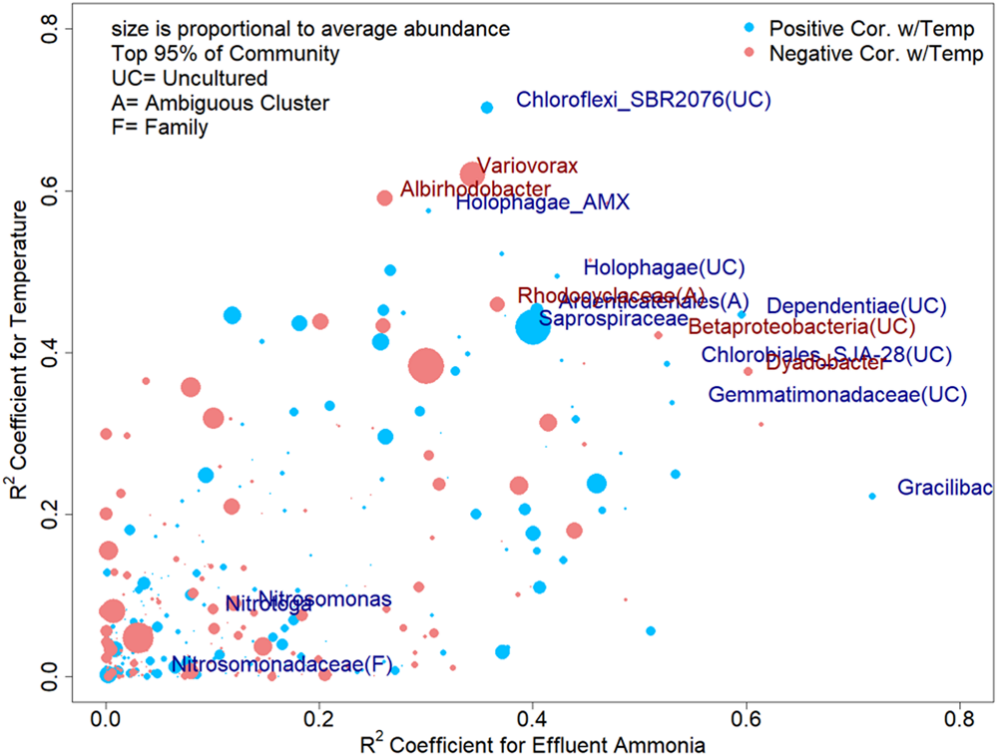
Correlation coefficients of OTUs (97% sequence similarity level) with effluent ammonia concentration and wastewater temperature for the entire sampling year. The size of the dots is proportional to the average abundance of the respective OTU. OTUs represented by blue dots have a positive correlation with temperature while OTUs represented by red dots have a negative correlation to temperature. OTUs are names by their highest taxonomic rank. OTU names are given for all OTUs with correlation coefficients of >0.4 to either wastewater temperature or effluent ammonia concentration except for OTUs classified as belonging to taxa comprising known nitrifiers in order to emphasize their weak correlation with both of these parameters. A complete list of all highlighted OTUs in this figure can be found in Table S5 in the Supplementary Information.

We identified OTUs that changed in relative sequence abundance in correlation to annual fluctuations of wastewater temperature and effluent ammonia concentrations by calculating Pearson’s product moment correlation coefficients (R^2^) between OTU abundance and each of the two other variables. The two correlation coefficients (for water temperature and effluent ammonia concentrations) were plotted against each other for the top 95% most abundant OTUs of the activated sludge microbial community (Fig. 4). In Fig. 4, all OTUs with correlation coefficients of R^2^ > 0.4 for either variable, wastewater temperature or effluent ammonia concentration, and a relative sequence abundance of >0.1% are labeled with their specific taxa names.

Among the core community OTUs, an uncultured *Saprospiraceae* (7.64% ± 2.9%), a *Comanonadaceae* (*Variovorax* (3.38 ± 2.0%)), a *Rhodocyclaceae* (0.92% ± 0.6%), and the two uncharacterized taxa of *Chlorobi* (classified as *Chlorobiales* SJA-28 (0.14 ± 0.1%)) and *Gemmatimonadaceae* (0.12 ± 0.2%) correlated highest with the observed changes in wastewater temperature and effluent ammonia concentrations (either of the two correlation coefficients >0.4).

Several OTUs with high correlation coefficients to wastewater temperature and effluent ammonia concentrations did not occur year-round but were instead only present for multiple consecutive seasons. Among these OTUs an uncultured *Choroflexi* SBR2076, a *Cytophagaceae* (*Dyadobacter*), and an uncultured TM6 (*Dependentiae*) occurred in two or three consecutive seasons at relative sequence abundances ranging from 0.49 ± 0.5%, 0.28 ± 0.4%, to 0.01 ± 0.0%, respectively. Another uncharacterized taxa of *Chloroflexi* further classified as *Ardenticatenales* occurred only in the fall of 2015 (single season OTU) with a relative sequence abundance of 0.57 ± 0.6% but correlation coefficients >0.4 to wastewater temperature and effluent ammonia concentrations (R^2^ = 0.45 and R^2^ = 0.40, respectively). Also some of the only transiently occurring OTUs showed strong correlation with shifts in wastewater temperature and effluent ammonia concentrations. These OTUs comprise the *Alphaproteobacteria Albirhodobacter* (0.92 ± 0.6%), an uncultured marine betaproteobacteria hot creek clone (0.22 ± 0.2%), an uncharacterized *Gracilibacteria* (0.01 ± 0.0%), and two *Acidobacteria*, *Holophagae* belonging to subgroup 7 (0.12 ± 0.2%) and subgroup 10 (0.13 ± 0.2%). Transient OTU were generally of low relative sequence abundance.

Interestingly, none of these OTUs, belonging to any taxa of known nitrifying bacteria in our activated sludge sequence data (e.g. the family *Nitrosomonadaceae*, or the genera *Nitrosomonas* and *Nitrotoga*), met these criteria, indicating that the relative sequence abundance of known nitrifiers did not significantly change with the observed seasonal variations in water temperature and effluent ammonia concentrations (Fig. 4). Also see the corresponding quantitative PCR results for the ammonia monooxygenase gene (*amoA*) below.

The three most abundant OTUs in each of the four arbitrary OTU groups (core, single season, multiple season, transient) are shown Fig. S4 in the Supplementary Information in order to emphasize that most of the abundant OTUs in each category did not show a strong correlation (R^2^ < 0.4) with either wastewater temperature nor effluent ammonia concentration. The sum of the relative sequence abundance of the top three most abundant core OTUs averaged 20.7% ± 2.2%, while multiple season OTUs had a cumulative abundance of 1.3% ± 0.6%, and single season OTUs 0.3% ± 0.1%.

**Figure 5:**
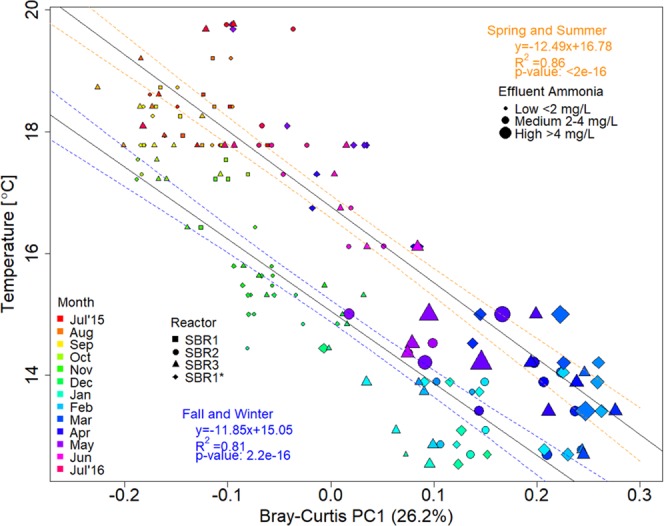
Linear regression of the first principle component (PC1) of the Bray-Curtis dissimilarity analysis with the observed fluctuation in wastewater temperature. The plot shows all 55 weekly samples of each of the three triplicate reactors (squares, circles, triangles, diamonds). Reactor 1 samples are shown before (squares) and after (diamonds) impellor failure at week 18 (November 2015). The size of each symbol denotes the effluent ammonia concentration. The rainbow-color scheme represents the sampling months with warmer summer and fall months in red/yellow colors and colder spring and winter months in blue/green colors. Linear regression lines with 95% confidence intervals for the cooling seasons (fall and winter; blue) and warming seasons (spring and summer; orange) show the strong correlation of microbial community richness with the recorded changes in wastewater temperature throughout the sampling year.

In order to quantify the compositional dissimilarity of the activated sludge microbial community between all 55 weekly samples of each of the three reactors, we calculated Bray-Curtis, Euclidian, and Kulczynski metrics and performed principle coordinates analysis to visualize (dis)similarities among the 16S rRNA gene amplicon data for each sample^37,43,44^. All statistical dissimilarity matrices revealed similar results (See Figs S5, S6a–d in Supplementary Information) showing distinct seasonal shifts among the activated sludge communities for all three reactors. Samples collected during the winter months (blue colors) and the summer months (red colors) cluster at opposite ends of the first principle coordinate that explains most of the variation in the dataset. In Fig. 5 the first principle coordinate for the Bray-Curtis metrics is plotted against the seasonal change in wastewater temperature emphasizing the distinct shift in microbial community composition with seasonal variation in wastewater temperatures (see also Fig. S12 for the portion of the total variance explained by Bray-Curtis dimensions). The fall and winter months, when wastewater temperature gradually declined, and the spring and summer months, when wastewater temperatures gradually increased again, both revealed a statistically significant (p < 2e-16), strong linear correlation (R^2^ > 0.81) of microbial community dissimilarity with the seasonal trends in wastewater temperature change. Interestingly, the linear regression did not show significant differences between the slopes (Ancova p < 0.948) of the two regression lines indicating that the rate of temperature change during fall/winter ‘cool-down’ and spring/summer ‘warm-up’ was constant. However, the ‘cool-down’ and ‘warm-up’ regression lines had significantly different y-intercepts (Ancova p < 2.2e-16) revealing a temperature gap of about 1.7 °C between winter ‘cool-down’ and summer ‘warm-up’. As consequence of the temperature offset between wastewater ‘cool-down’ starting fall 2015 and ‘warm-up’ beginning in spring 2016 the microbial community composition in the activated sludge of the sampled plant were significantly different from each other in summer 2015 and summer 2016 (Fig. S10 in Supplementary Information).

Using other matrices to quantify the compositional dissimilarity of the activated sludge microbial community showed that the temperature gap between seasonal cooling and warming periods was remarkably invariant, with 1.7 °C and 1.1 °C for the Kulczynski and Euclidean dissimilarity matrices, respectively (Fig. S6a–d, in the Supplementary Information).

### **Known functional guilds involved in N-cycling**

#### *Taxa known to comprise ammonia- and nitrite-oxidizing bacteria*

**Figure 6:**
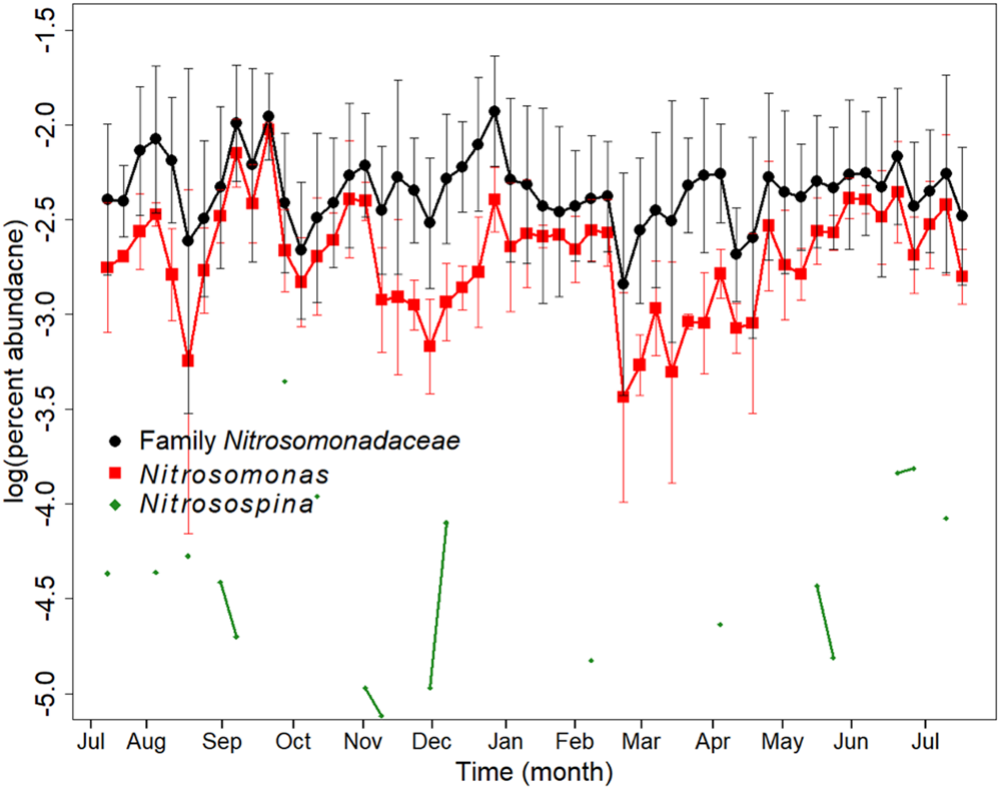
Average relative sequence abundances (three parallel reactors) of OTUs affiliated to taxa comprising known ammonia-oxidizing bacteria identified in the activated sludge samples from the wastewater treatment plant at Brainerd, MN, between July 2015 and July 2016. Green *Nitrosospina* OTUs, red *Nitrosomonas* OTUs, and black sum of all *Nitrosomonadaceae* OTUs (including an ambiguous OTU cluster affiliated to this family). While *Nitrosomona*s have been detected in all samples throughout the entire year, *Nitrosopina* have only been detected sporadically in one of the three sequencing batch reactors.

We searched the activated sludge 16S rRNA gene amplicon data for taxa known to contain microorganisms capable of ammonia oxidation and nitrite oxidation. The known ammonia-oxidizing bacteria in our activated sludge samples all belonged to the family of *Nitrosomonadaceae*, comprising the genera *Nitrosomonas* and *Nitrosospina*. Only *Nitrosomonas* were detected year-round representing on average between 0.2 and 0.5% of the core community. *Nitrosospina* belonged to the category of transient OTUs because they were only detected sporadically (Fig. 6). Interestingly, we also found anaerobic ammonia-oxidizing bacteria belonging to Candidatus *Brocadia* of the Planctomycetales in some of the samples. The *Brocadia* OTU we identified was the only known ammonia-oxidizing taxa that reveal a significant correlation to changes in wastewater temperature. While the overall sequence abundance of Candidatus *Brocadia* was relatively low (<0.03%), the taxa increased 2.5-fold in relative sequence abundance between the months of January and July when temperatures dropped from 15 °C to 12.2 °C in winter 2015/spring 2016 (Fig. S7 in the supplemental information).

Known nitrite-oxidizing bacteria belonged to *Nitrotoga*, *Nitrospira*, and *Nitrobacter*. *Nitrotoga* belonged to the core community detected throughout the entire year at a relative sequence abundance of on average >0.5%. *Nitrospira* and *Nitrobacter* have only been identified in some of the weekly samples. Both taxa occurred sporadically and have not been detected as part of the activated sludge community over an entire season. The relative abundance of the complete nitrifying microbial community, comprising known taxa of ammonia and nitrite-oxidizing bacteria was 0.96% ± 0.37%. This relative abundance is within the lower range of typical reported abundances for nitrifier communities in activated sludge samples^21,23,26,45^. For example, Griffin *et al*.^21^ report a relative abundance of *Nitrosomonas* of ~0.5%, while the relative abundances of *Nitrospira* varied between 0.75% to 2.3% in their system. However, Ju *et al*.^16^ and Saunders *et al*.^26^ reported a relative sequence abundance for *Nitrosomonas* of less than 1%. None of the known nitrifying functional guilds we detected in our activated sludge samples correlated significantly to either changes in wastewater temperature and effluent ammonia concentrations. Also see Fig. 3 and description of results above.

#### *Functional marker genes of ammonia-oxidizing bacteria and archaea*

**Figure 7:**
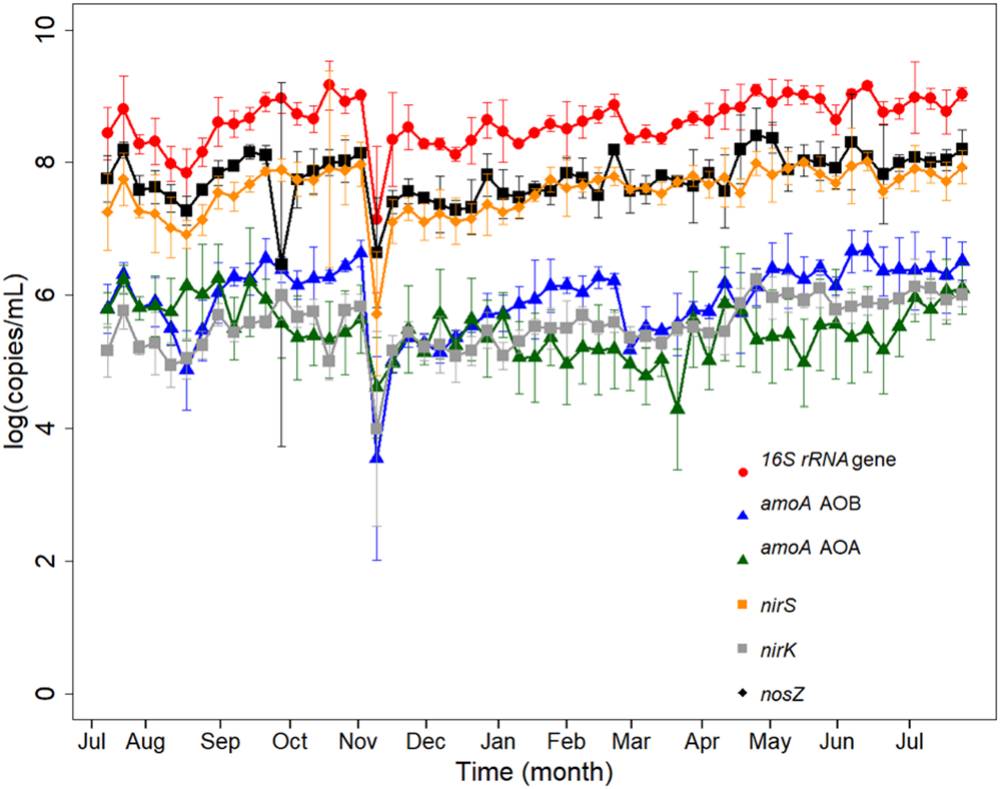
Quantitative PCR results showing the change in gene copy numbers over time for the 16S rRNA gene and process-relevant functional marker genes for microbial nitrification and denitrification. Gene copy number are show as log copy number per milliliter of activated sludge. Gene copy numbers of the 16S rRNA gene (red circles), ammonia monooxygenase gene of ammonia-oxidizing bacteria (*amoA* gene of AOB; blue triangles), ammonia monooxygenase gene of ammonia-oxidizing archaea (*amoA* gene AOA; green triangles), nitrite reductase *nirS* (orange squares), nitrite reductase *nirK* (gray squares), nitrous oxide reductase *nosZ* (black diamonds). All gene copy numbers show a significant decrease at week 18 in November 2015 when the impellor or reactor 1 broke and had to be replaced. Apart from this incidence gene copy numbers of all quantified genes were relatively stable and no statistically significant changes (Anova p < 0.05) were identified throughout the entire year.

We used quantitative PCR to monitor the abundance of the 16S rRNA gene and several functional marker genes of key microbial nitrogen transformation processes in activated sludge such as ammonia oxidation, nitrite reduction, and nitrous oxide reduction. In order to quantify ammonia-oxidizing Bacteria and Archaea we used primers targeting the functional marker gene ammonia monooxygenase (*amoA*). Denitrifying bacteria were quantified by targeting the two nitrite reductase genes *nirS* and *nirK*, while microorganisms capable of nitrous oxide reduction were quantified targeting the nitrous oxide reductase gene *nosZ* clade 1. The quantitative PCR results are summarized in Fig. 7. The synchrony of the qPCR data for reactors 2 and 3 was highly significant for all quantified genes over the entire year. Reactor 1 was distinct from reactors 2 and 3 with respect to *nosZ* gene copy numbers (p = 0.8686 and p = 0.7901) and distinct from only reactor 3 for *nirK* gene copy numbers (p = 0.5181), which might be due to the impellor failure of reactor 1 at week 18.

Over the entire year of sampling average 16S rRNA gene copy numbers were 8.62 ± 0.37 log(copies/mL). Shifts in 16S rRNA gene abundance during the year did not correlate with wastewater temperature and effluent ammonia concentrations. Overall, 16S rRNA gene copy numbers were three orders of magnitude higher than both bacterial and archaeal *amoA* gene copy numbers. Average annual *amoA* gene copy numbers were 5.94 ± 0.56 log(copies/mL) for Bacteria and 5.47 ± 0.42 log(copies/mL) for Archaea. A linear regression of bacterial *amoA* gene copy numbers with the cumulative 16S rRNA gene sequence abundance of all OTUs classified as *Nitrosomonadaceae* over the entire years resulted in a correlation coefficient of R^2^ 0.64 (p-value 5.21e^−13^) (Fig. S9). The strong and significant correlation of both datasets confirmed that the population abundance of known ammonia-oxidizing bacteria is relatively stable throughout the year and does not change significantly when wastewater temperature dropped in the winter month.

We also tried to quantify complete ammonia oxidizing bacteria affiliated to the Nitrospira (Comammox Nitrospira) by using the recently published *amoA*-targeted qPCR primers of Pjevac *et al*.^46^ (data not shown). In almost all activated sludge samples collected in this study comammox Nitrospira were below our qPCR assay detection limit.

#### *Functional marker genes of denitrifying microorganisms*

The abundance of the nitrite reductase gene *nirS* was two orders of magnitude higher than the abundance of the *nirK*-type nitrite reductase. We quantified 7.57 ± 0.39 log(copies/mL) for the *nirS*-type nitrite reductase and only 5.55 ± 0.39 log(copies/mL) for the *nirK*-type nitrite reductase. Clade I-type nitrous oxide reductase gene (*nosZ*) copy numbers were 7.81 ± 0.47 log(copies/mL) (Fig. 7). All functional genes quantified by qPCR did not show a significant change in abundance with the observed decrease in wastewater temperature and the corresponding increase in effluent ammonia concentration during the winter season.

## Discussion

### Reactor synchrony

At first, we compared the three parallel reactors with each other to evaluate if they are true replicates or show any significant differences with respect to composition and structure of their activated sludge communities. In this context it is important to consider that the WWTP in Brainerd, MN does not have an upstream equalization basin or primary settling tank to homogenize influent wastewater. Interestingly, despite constant re-seeding of reactors with influent wastewater every 6 hours (average duration of a reactor cycle) we did not find significant differences in alpha diversity between the three reactors at any time during the year-long sampling campaign. Only on week 18, when the impellor of reactor 1 broke, differences in relative amplicon abundance and qPCR gene copy numbers became significant for reactor 1 compared to the other two reactors. While maintenance work required drastic changes in sludge volume of reactor 1 during week 18, reactor re-start by using activated sludge from the two other operational reactors established a microbial community with insignificantly different alpha diversity to the other two reactors within a week. We therefore decided to eliminate the week 18 sample of reactor 1 from the dataset and combine samples of week 1 to 17 and 19 to 55 into one continuous set of data for reactor 1. The observed similarity between the independent triplicate reactors is interesting because it means that any so slight variations in the chemical composition, physical parameters, and community composition of the influent wastewater over time, did not change the activated sludge microbial community in any of the three reactors in a way that were statistically significant. Any minor shifts in community composition between reactors resulted from transient OTUs that occurred for only a short period of time at low relative sequence abundance before been washed out of the system again (most OTUs were singletons). The high similarity between reactors was also supported by the fact that process performance did not significantly alter between reactors at any time during the sampling year. Overall, core and shared seasonal OTUs accounted for 87.7% and 93.3% of the activated sludge communities in the three reactors. A higher similarity of the activated sludge microbial communities within different reactors of the same full-scale WWTP than between different plants has also been observed in previous studies^21^.

From the perspective of an operator of a wastewater treatment plant similar to the facility in Brainerd, the similarity in community composition and structure among the three parallel reactors is an important finding because reactor failures, such as the technical problems observed in reactor 1 at week 18, will not affect the other reactors nor impair overall plant performance. The operational reactors can serve as seed banks for inoculation when maintenance or technical issues require a restart of one reactor.

### **Seasonal community dynamics**

Activated sludge communities have been reported to be shaped by both deterministic and neutral factors^21,26–30^. At the Brainerd WWTP, temporal variation in community structure was primarily driven by changes in wastewater temperature (Fig. 3). Also, previous studies have reported on strong seasonal effects associated with temperature on the bacterial community composition and structure in WWTP^21,47^.

Griffin and Wells^21^ recently reported that the community dynamics in six full-scale reactors at four WWTPs in the Chicago area (Illinois, USA) displayed a highly reproducible and synchronous seasonal fluctuation. They also observed that after one complete year of sampling individual reactors maintained minor but stable differences in community composition. We also found that the summer 2015 and summer 2016 microbial communities at the Brainerd WWTP in Minnesota were significantly different from each other (Fig. S10), however, both summer sample were more similar to each other than to any of the samples from the other three seasons (winter, spring, and fall) (Fig. S5). This suggests a seasonal community succession but continual annual drift of the activated sludge community at the Brainerd facility. How this annual community drift will affect plant functional stability and process performance on a long-term annual time scale will be an interesting topic for future studies. Based on our current data it is not possible to discern seasonal trends within one year from log term variations between consecutive years. However, seasonal community succession across multiple years has been describes as a distinct characteristic of aquatic microbial communities in freshwater and marine habitats^48–50^. For activated sludge systems seasonal temperature fluctuations seem to be mainly a predominant deterministic factor for the composition of communities of WWTP located in temperate climate zones, while WWTP in tropical climates with warmer, less variable average annual temperatures, continual community drift rather than seasonal succession patterns seems to drive variation in community composition^16^. Interestingly, the microbiomes in WWTP located in polar regions with continuous cold climate seem to be able to maintain high performance levels in terms of organic matter and nutrient removal but these plants are usually operated at SRTs of up to 30 days^22^.

Because temperature seems to be a major driver of seasonal community succession at the Brainerd WWTP, the significant differences between the summer 2015 and 2016 activated sludge communities might be explainable by the observed temperature gap between cooling and warming periods. While the rate of temperature change during cooling and warming phases was similar (±0.043 °C/day) recovery of alpha and beta diversity in summer 2016 was delayed by 5.1 SRT cycles (calculation based on an annual average of 7.8 days per SRT) compared to activated sludge microbial diversity described in summer 2015. More data will be need to evaluate if this observation is a reproducible characteristic causing annual drift of activated sludge communities in sequencing batch reactors of temperate climate WWTPs.

Co-occurrence networks can help to identify interconnections between OTUs based on their paired presence within complex communities^51^. A co-occurrence analysis performed on the dataset obtained in this study did not identify any meaningful interactions between community members (data not shown) because of the high abundance of a relative low number of core OTUs with many interactions which reduced the interpretability of the obtained network. This is a known limitation of networks that are characterized by local hot spots of abundant correlations between neighboring network notes^52^. Considering that variable copy number of ribosomal RNA genes can influence relative OTU abundances in sequencing libraries and the fact that metabolic functions are generally not well conserved among ribotypes, does further limit the meaningful interpretation of co-occurrence networks for the identification of putative interactions between microorganisms in the environment.

### Nitrifying community

The WWTP in Brainerd repeatedly experienced a loss in nitrification performance when wastewater temperatures decreased below 13 °C during the cold Minnesota winters. Despite the observed decrease in N-removal efficiency our sequencing and qPCR results showed that the abundance of known nitrifying microbial populations was rather constant throughout the entire year. As mentioned above lower temperatures reduce microbial growth rates and may wash out slow-growing nitrifiers if sludge retention times are kept constant and relatively short (annual average 7.8 days) all year long. Under this assumption we expected that the decrease in nitrification performance in the winter month might be associated with a decrease in nitrifier population abundance. However, our 16S rRNA gene amplicon sequencing and qPCR data did not reveal any significant correlation between relative OTU abundances, ammonia monooxygenase gene copy numbers and the observed seasonal fluctuations in temperature and effluent ammonia concentration. This means that the nitrifying community must maintain a growth rate higher than the inverse of the SRT in order to remain in the system. However, at the same time we observed a decrease in plant nitrification performance which means that the populations of known nitrifiers maintained their growth rates while lowering their energy generation via ammonia oxidation. Maintaining growth rates at lower rates of ammonia oxidation might be possible when organisms are metabolically versatile and flexible and can switch over to another energy metabolism or when immigration from influent sources maintain a stable population abundance. It is also conceivable that other species than the typical, known ammonia oxidizers contribute to the ammonia oxidation rates in the warmer spring and summer months but that these unknown “seasonally active” microorganisms are temperature sensitive and are contributing less to the observed amount of ammonia removal in the winter. The fact that ammonia removal activity is never completely lost and activity is recovered in spring means that known and unknown microbial taxa capable of ammonia-oxidation must remain in the reactors year-round or are continuously immigrating with influent wastewaters.

Other studies that report on the correlation of seasonal nitrification failure with ammonia-oxidizing community dynamics based on 16S rRNA gene amplicon sequencing also observed that nitrifying populations can maintain a relatively stable abundance despite seasonal fluctuations in wastewater temperature^21^. Delatolla *et al*.^19^ quantified the biomass and rRNA content in a wastewater biofilm by fluorescence *in situ* hybridization and microscopy and did not find evidence for seasonal effects on the abundance of nitrifying microorganisms at colder temperatures^19^. Conversely, Beneduce *et al*.^13^ showed that the abundance of ammonia-oxidizing bacteria was severely affected by drastic changes in temperature, dissolved oxygen, and salinity in a wastewater treatment plant treating water from a saline thermal spa.

More data might be necessary to identify if the observed change in nitrifying activity is solely thermodynamically driven^17^, or a consequence of microbial populations entering or leaving the system throughout the seasons. In Fig. 4 we identified a couple of OTUs that showed a strong correlation in relative sequence abundance with either temperature and/or effluent ammonia concentration (also see Table S5 in Supplementary Information). An OTU classified as uncultured *Saprospiraceae* was the second most abundant OTU in all three SBRs with an average annual abundance of 7.64% ± 2.9%. We observed a significant positive correlation with temperature as well as a significant negative correlation with effluent ammonia concentration (Pearson R = 0.69 & 0.63, p < 8.1e-9 & 4.6e-5) while the relative sequence abundance of this *Saprospiraceae* OTU ranged from 12.0% during the summer down to 3.0% in the winter. We also identified a second *Saprospiraceae* OTU, classified as *Candidatus Aquirestis*, as one of the top three most abundant OTUs in the ‘multiple seasons’ OTU category since we detected it only in Fall of 2015 and Spring and Summer 2016 but not during the winter month. Important in this context might be that it has been shown in previous studies that with the decrease in temperatures the structure and species composition of microbial aggregates and biofilms in activated sludge can change which may directly or indirectly impact nitrification performance^19,20^. *Saprospiraceae* have been described as epiphytic rods that attach to filamentous bacteria^53^. They are commonly found in flocs and aggregates of activated sludge. However, their exact role in wastewater treatment is not well understood^21,24,25,54,55^. Members of the *Saprospiraceae* may be important in the breakdown of complex carbon sources. It has been suggested that their hydrolytic activity provides simple carbon substrates for other microorganisms involved in the removal of nutrients and this might even be the rate-limiting step for these processes^56,57^. However, most nitrifiers are autotrophs and do not rely on organic carbon for growth. On the other hand, Reza and Alvarez Cuenca^58^ suggested that members of the family *Saprospiraceae* could also be directly involved with the nitrification processes.

It might be conceivable that a change in abundance of *Saprospiraceae* during the winter has an effect on the structural integrity of activated sludge aggregates which will alter the habitat space of nitrifier populations. This might not directly affect nitrifier population abundances but could indirectly affect nitrification efficiency if ammonia oxidizers lose the structural support for forming close spatial associations with nitrite oxidizers in activated sludge flocs. However, in order to test this hypothesis a microscopic study will be required to visualize and quantify the involved microbial taxa and their spatial association in accordance with seasonal extremes of high and low wastewater temperatures. In this context future studies should also evaluate the role of the ambiguous taxonomic groups affiliated with the phyla *Chlorobi* and *Chloroflexi* (Table S5) that we identified as core or multiple season taxa with significant correlation to wastewater temperature and effluent ammonia concentrations, for their contributions to aggregate stability and reactor nitrogen removal processes.

## Conclusions

We investigated the composition and seasonal dynamics of activated sludge communities of three full-scale sequencing batch reactors of a wastewater treatment plant in a temperate climate zone, with focus on changes in community structure in response to environmental variables. We showed that:

- Temperature was the primary driver of shifts in alpha and beta community diversity
- Seasonal patterns of microbial community structure exist in highly synchronized reactors
- A set of core OTUs were highly abundant and strongly synchronized between reactors
- Individual OTUs only occurred seasonally (single season, multiple seasons) or for shorter periods of time in any of the three reactors
- Key functional groups such as ammonia oxidizing bacteria maintained stable population abundances despite decrease in plant ammonia removal performance in the winter month

To date this study is one of the most comprehensive analyzes of seasonal community dynamics in the activated sludge microbiome of SRBs in temperate climate zones that experience a periodic decrease in nitrogen removal efficiency. A more in-depth understanding of the seasonal community composition and dynamics will be beneficial for treatment plants which continually struggle to maintain efficient nitrification activities despite following typical mitigation strategies such as prolonging SRT and increasing total biomass. Future time series studies should assess the transcriptional activity of nitrification-associated genes, in order to quantify microbial activity shifts associated with cold temperature nitrification failure so that factors controlling the abundance of nitrifying populations can be better understood. The data presented here will help to develop predictive models and new concepts in order to improve bioreactor design and operation condition for wastewater treatment plants in temperate climate zones so that they can achieving reliable nitrification performance at all seasons.

## Supplementary information

Supplementary Information

## Data Availability

Illumina sequencing reads have been deposited in the National Center for Biotechnology Sequence Read Archive under Accession Number PRJNA490320.
