# Supplementary material for: Composition and Dynamics of the Activated Sludge Microbiome during Seasonal Nitrification Failure
Source: Sci Rep. 2019 Mar 14;9:4565. doi: 10.1038/s41598-019-40872-4 (PMC6418219; doi:10.1038/s41598-019-40872-4)
Supplement: Supplementary file 1 — Supplementary Information [file 41598_2019_40872_MOESM1_ESM.docx]

**Composition and Dynamics of the Activated Sludge Microbiome during Seasonal Nitrification Failure**

Juliet Johnston (she/her)^1^, Timothy LaPara (he/him)^1,2^, Sebastian Behrens (he/him)^1,2,*^

^1^ University of Minnesota, Department of Civil, Environmental, and Geo-Engineering, 500 Pillsbury Drive S.E, Minneapolis, MN 55455-0116, USA

^2^ University of Minnesota, BioTechnology Institute, 1479 Gortner Avenue, St. Paul, MN 55108-6106, USA

**- Supplementary Information -**

***Corresponding Author:**

Sebastian Behrens

University of Minnesota

BioTechnology Institute

Snyder Hall #338

1479 Gortner Avenue

St. Paul, MN 55108-6106

Phone: (612) 624-8454

e-mail: [sbehrens@umn.edu](mailto:sbehrens@umn.edu)

**Table S1.** Physical and chemical parameters of influent and effluent wastewater at the Brainerd wastewater treatment plant throughout the sampling year from July 2015 to July 2016.


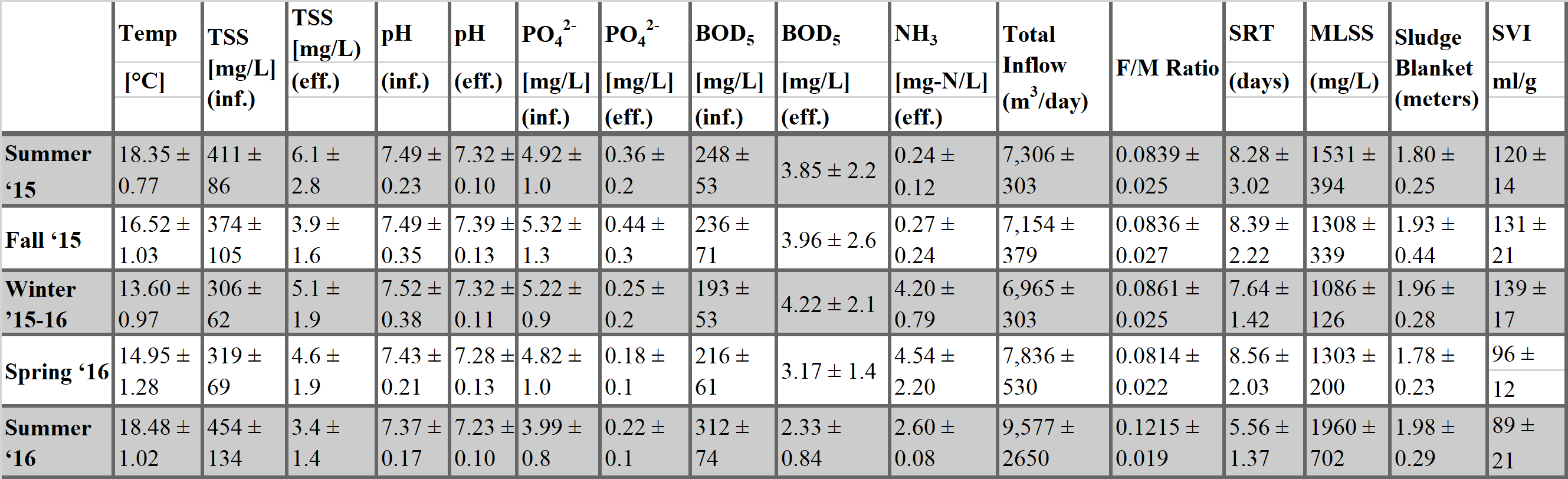


**Table S2.** Primer sequences used for quantitative PCR.

| **Target Gene** | **Forward Primer Sequence (5’- 3’)** | | **Reverse Primer Sequence (5’-3’)** | | **Reference** |
| --- | --- | --- | --- | --- | --- |
| ***16S rRNA gene* (V3 region)** | **338-F**  CCTACGGGAGGCAGCAG | **518-R**  ATTACCGCGGCTGCTGG | | (Nübel & Muyzer, 1997) | |
| ***amoA* (Bacteria)** | **550-F**  TCAGTAGCYGACTACACMGG | **745-R**  CTTTAACATAGTAGAAAGCG | | (Harms et al. 2003) | |
| ***amoA* (Archaea)** | **GenAOA-F**  ATAGAGCCTCAAGTAGGAAAGTTCTA | **GenAOA-R**  CCAAGCGGCCATCCAGCT GTATGTCC | | (Meinhardt et al. 2015) | |
| ***nirK*** | **nirK876c**  ATYGGCGGVCAYGGCGA | **nirK1040**  GCCTCGATCAGRTTRTGG | | (Braker et al. 1998) | |
| ***nirS*** | **nirSCd3aF**  AACGYSAAGGARACSGG | **nirSR3cd**  GASTTCGGRTGSGTCTTSAYGAA | | (Throback et al. 2004) | |
| ***nosZ Clade 1*** | **nosZ2F**  CGCRACGGCAASAAGGTSMSSGT | **nosZ2R**  CAKRTGCAKSGCRTGGCAGAA | | (Harter et al. 2017) | |
| ***amoA clade A***  ***(Comammox)*** | ***comaA-244F***  ***TAYAAYTGGGTSAAYTA*** | ***comA-659R***  ***ARATCATSGTGCTRTG*** | | (Pjevac et al., 2017; Throback et al., 2004) | |
| ***amoA clade B***  ***(Comammox)*** | ***comaA-244F***  ***TAYTTCTGGACRTTYTA*** | ***comA-659R***  ***ARATCCARACDGTGTG*** | | (Pjevac et al., 2017; Throback et al., 2004) | |

**Table S3.** P-values of a pairwise comparison of a linear regression of Shannon, Simpson, and Chao1 diversity indices for the activated sludge microbial communities in the sequencing batch reactors of the sampled WWTP. P-values > 0.05 indicate that the diversity indices for the reactor pairs are not significantly different, rejecting the null hypothesis that the slope is zero.

**Table S4** P-values of a t-test comparing the means of Shannon, Simpson, and Chao1 diversity indices for the activated sludge microbial communities in the sequencing batch reactors of the sampled WWTP for the four seasons. P-values < 0.05 indicate that the diversity indices for the respective season are significantly different for the respective reactor pair. Almost every season in all reactors had significantly different alpha diversities.

**Table S5**. OTUs with relative sequence abundance > 0.1% and Pearson's product moment correlation coefficients R^2^ > 0.4 for either of the variables wastewater temperature or effluent ammonia concentration. OTU classification at the 97% sequence similarity cut-off (species-level). The last column to the right shows if the respective taxa belongs to the OTU category core, single season, multiple season, or transient.

| OTU | R^2^ Temperature | R^2^ Effluent Ammonia | Average Abundance [%] | Season category |
| --- | --- | --- | --- | --- |
| *Bacteroidetes_Sphingobacteriia_Sphingobacteriales*  *_Saprospiraceae*_uncultured | 0.432 | 0.400 | 7.64% ± 2.92% | Core |
| *Proteobacteria_Betaproteobacteria_Burkholderiales*  *_Comamonadaceae_Variovorax* | 0.620 | 0.344 | 3.38% ± 2.08% | Core |
| *Proteobacteria_Alphaproteobacteria_Rhodobacterales*  *_Rhodobacteraceae_Albirhodobacter* | 0.591 | 0.261 | 0.92% ± 0.56% | Transient |
| *Proteobacteria_Betaproteobacteria_Rhodocyclales*  *Rhodocyclaceae_*ambiguous_taxa | 0.460 | 0.367 | 0.78% ± 0.49% | Core |
| *Chloroflexi_Ardenticatenia_Ardenticatenales*_ambiguous_taxa | 0.454 | 0.404 | 0.57% ± 0.58% | Single Season  Fall |
| *Chloroflexi*_SBR2076_uncultured bacterium | 0.702 | 0.357 | 0.49% ± 0.47% | Multiple Season  (Sum/Fall/Win) |
| *Bacteroidetes_Cytophagia_Cytophagales_Cytophagaceae*  *_Dyadobacter* | 0.377 | 0.602 | 0.28% ± 0.39% | Multiple Season  (Spr/Sum) |
| TM6 (*Dependentiae*)_uncultured bacterium | 0.447 | 0.596 | 0.01% ± 0.01% | Multiple Season  (Sum/Fall) |
| Proteobacteria _Betaproteobacteria _Hot Creek 32_  _uncultured marine bacterium | 0.422 | 0.518 | 0.22% ± 0.21% | Transient |
| *Gracilibacteria*_ambiguous_taxa | 0.223 | 0.718 | 0.01% ± 0.02% | Transient |
| *Chlorobi_Chlorobia_Chlorobiales*_SJA-28_uncultured bacterium | 0.387 | 0.525 | 0.14% ± 0.14% | Core |
| *Acidobacteria_Holophagae*_Subgroup 10_ABS-19 _bacterium enrichment culture clone | 0.576 | 0.302 | 0.13% ± 0.17% | Transient |
| *Gemmatimonadetes_Gemmatimonadetes_Gemmatimonadales*  *_Gemmatimonadaceae*_uncultured | 0.338 | 0.530 | 0.12% ± 0.18% | Core |
| *Acidobacteria_Holophagae*_Subgroup 7_uncultured soil bacterium | 0.495 | 0.423 | 0.12% ± 0.18% | Transient |
| OTU affiliated to taxa of known nitrifying bacteria | | | | |
| *Proteobacteria_Betaproteobacteria_Nitrosomonadales*  *_Nitrosomonadaceae_Nitrosomonas* | 0.127 | 0.085 | 0.24% ± 0.19% | Core |
| *Proteobacteria_Betaproteobacteria_Nitrosomonadales*  *_Nitrosomonadaceae* | 0.038 | 0.005 | 0.48% ± 0.26% | Core |
| *Proteobacteria_Betaproteobacteria_Nitrosomonadales*  *_Gallionellaceae_*Candidatus *Nitrotoga* | 0.101 | 0.079 | 0.47% ± 0.30% | Core |

**Table S6.** Using the quantitative PCR data to compare reactors these p-values were used determine a linear relationship between each SBRs’ gene abundance with an Anova test. Cells are highlighted in red when p<0.05. This was complimented with a Students t-test since most reactors did not change significantly throughout the year.

**Table S7.** Using the quantitative PCR data to compare reactors a Student’s t-test was performed to compare the means of the average gene abundance between reactors. Red cells highlight p <0.05. The average gene abundance throughout the year is not statistically different between reactors with a few exceptions when comparisons are made with SBR 1 and SBR 1* which were not operational throughout the entire year.

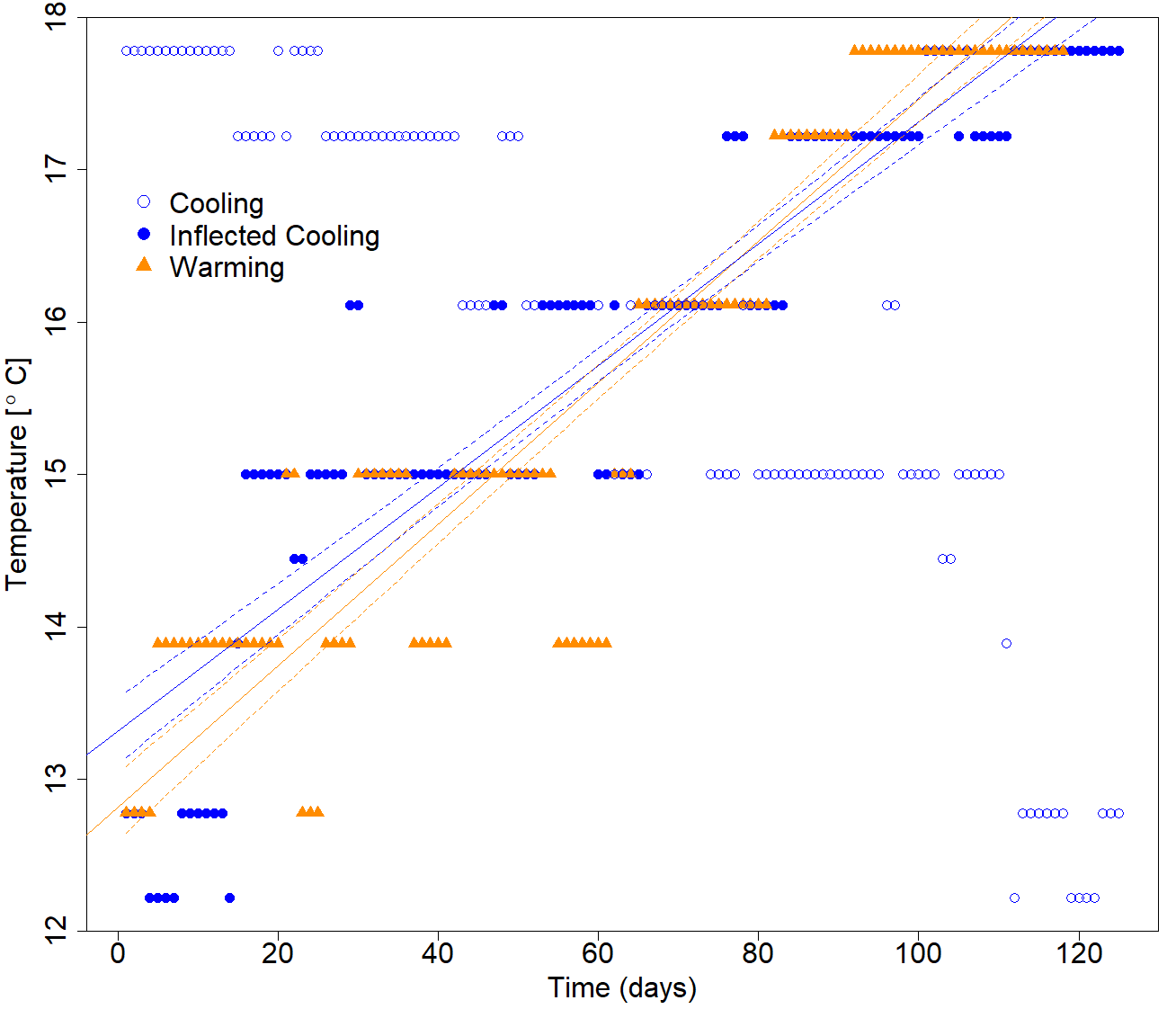


**Figure S1.** This plot denotes the temperature regression between Fall and Winter against Spring and Summer. The blue outlined circles show the original Fall and Winter data whereas the blue filled circles are inflected over the x-axis to show the flipped alignment alongside the orange triangle Spring and Summer data points. The slopes are not statistically different with the average rate of change as approximately ±0.043°C/day. The solids lines denote the linear regression while the dashed lines denote the 95% confidence interval for the average.


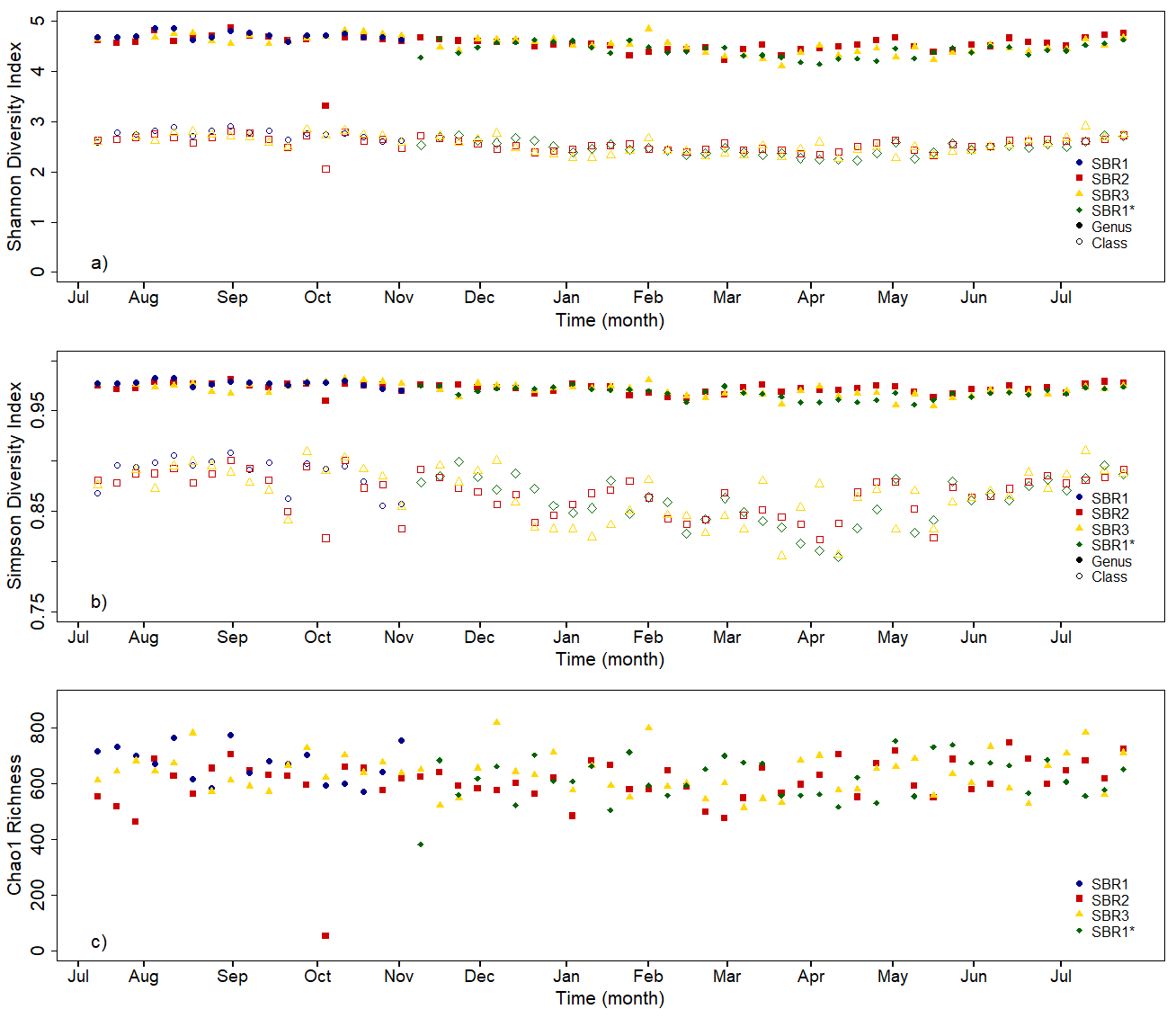


**Figure S2.** Different alpha diversity metrics were performed across the different reactors through a) Shannon Diversity b) Simpson Diversity, and c) Chao1 Richness. Each sequencing batch reactor is shown individually as blue circles for SBR1, red squares for SBR2, yellow triangles for SBR3, and green diamonds for SBR1*. Simpson and Shannon diversity were calculated at two levels, genus level (solid shapes) and class level (outlined shapes).

**Figure S3.** The average number of genus level OTUs in the core community (blue), seasonal community (both multiple and single season are in orange), and transient community (yellow) for each season. The black dashed line is exact values shared by all reactors while transient is an average between the reactors. The core community stays constant throughout the year, the transient community also remains consistent, while the seasonal OTUs decrease the richness of the reactors in the winter.


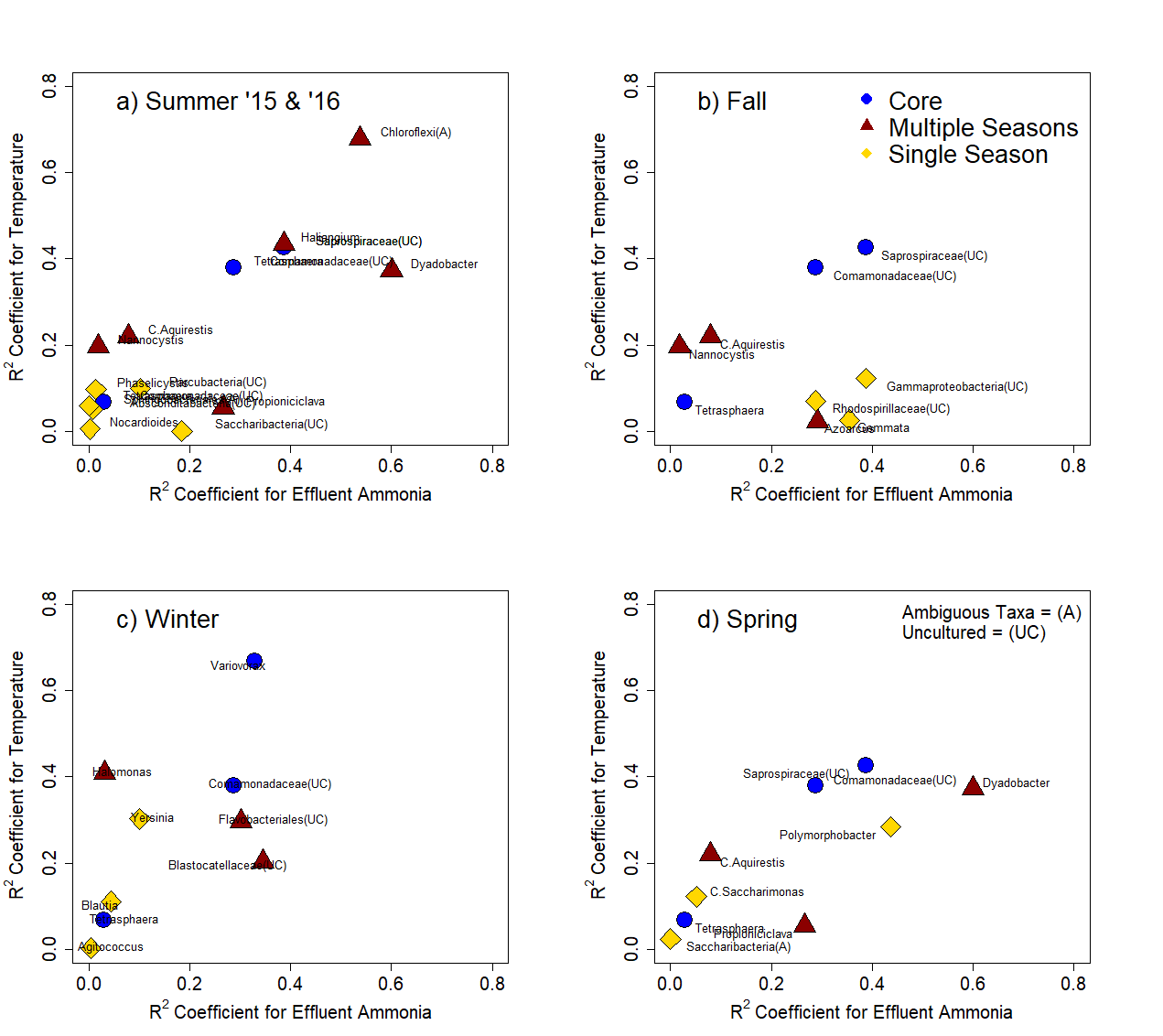


**Figure S4.** The correlation coefficients regressed against influent water temperature and effluent ammonia concentration are on the y and x-axis respectively for the top 3 highest abundance OTUs in the core (blue dots), multiple season (red triangles), and one season communities (yellow diamonds). These high abundance OTUs are often categorically seasonal as opposed to temperature dependent regressions.


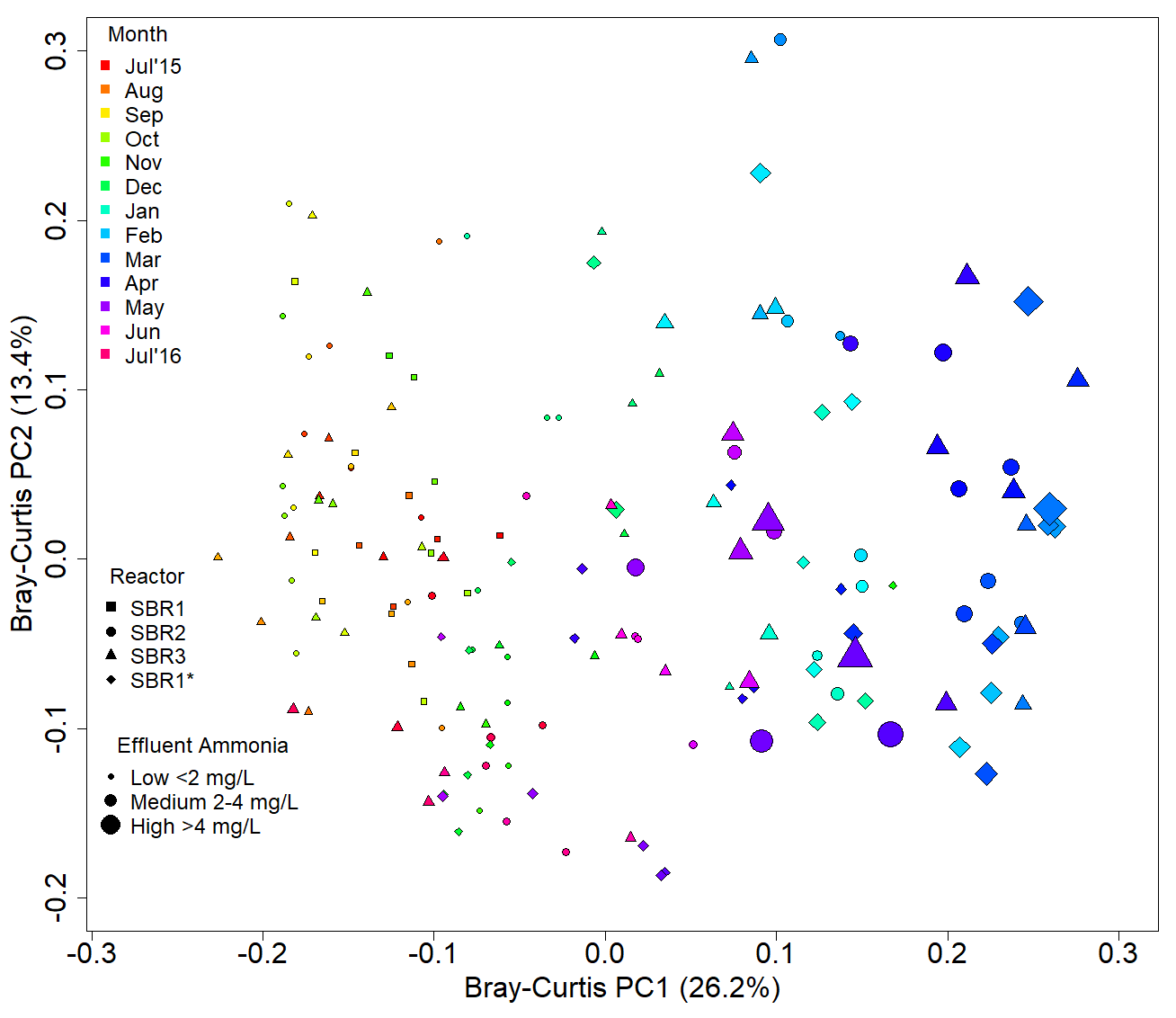


**Figure S5.** Principal Coordinates analysis was performed using Bray-Curtis dissimilarity. The rainbow color gradient denotes the changes in the months. Each reactor is plotted separately based on shape (Square-SBR1, Circle-SBR2, Triangle-SBR3, Diamond-SBR1*). The effluent ammonia concentration denotes the size of the data points. PC1 represents a separation based on the months and effluent ammonia concentration.


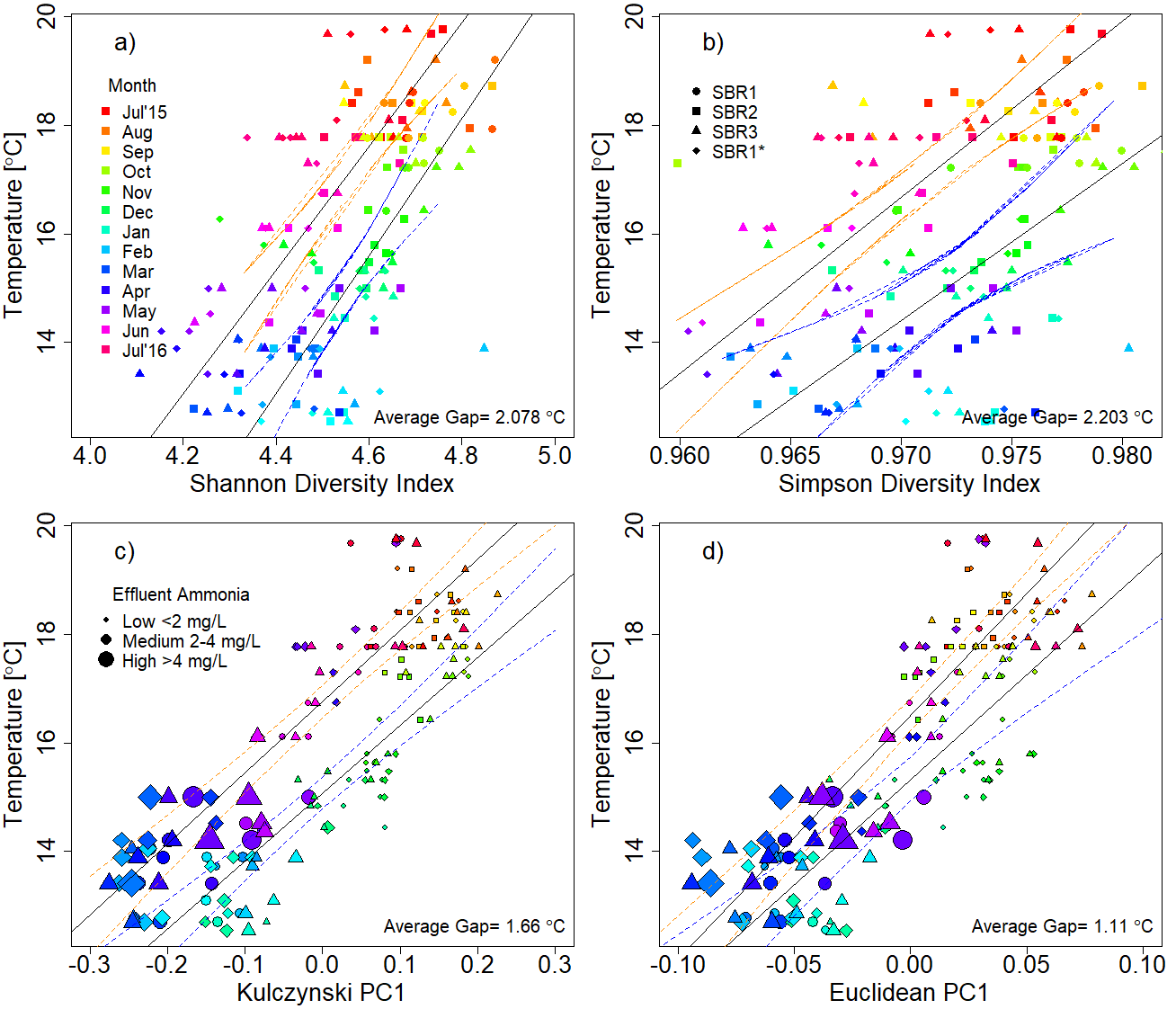


**Figure S6.** Several diversity metrics were regressed against the influent water temperature including a) Shannon diversity, b) Simpson diversity, c) Kulcynski, and d) Euclidean. The rainbow color gradient denotes the changes in the months. Each reactor is plotted separately based on shape (Square-SBR1, Circle-SBR2, Triangle-SBR3, Diamond-SBR1*). The linear regression is shown with the 95% confidence intervals as dashed lines for Spring and Summer in orange and Fall and Winter in blue. Each plot shows similar recovery lags ranging from 1.11°C up to 2.20°C. The first two figures represent this trend in a single dimension with alpha diversity while the other two represent this trend on the highest axis of variance in multi-dimensional, beta diversity dissimilarity matrices.


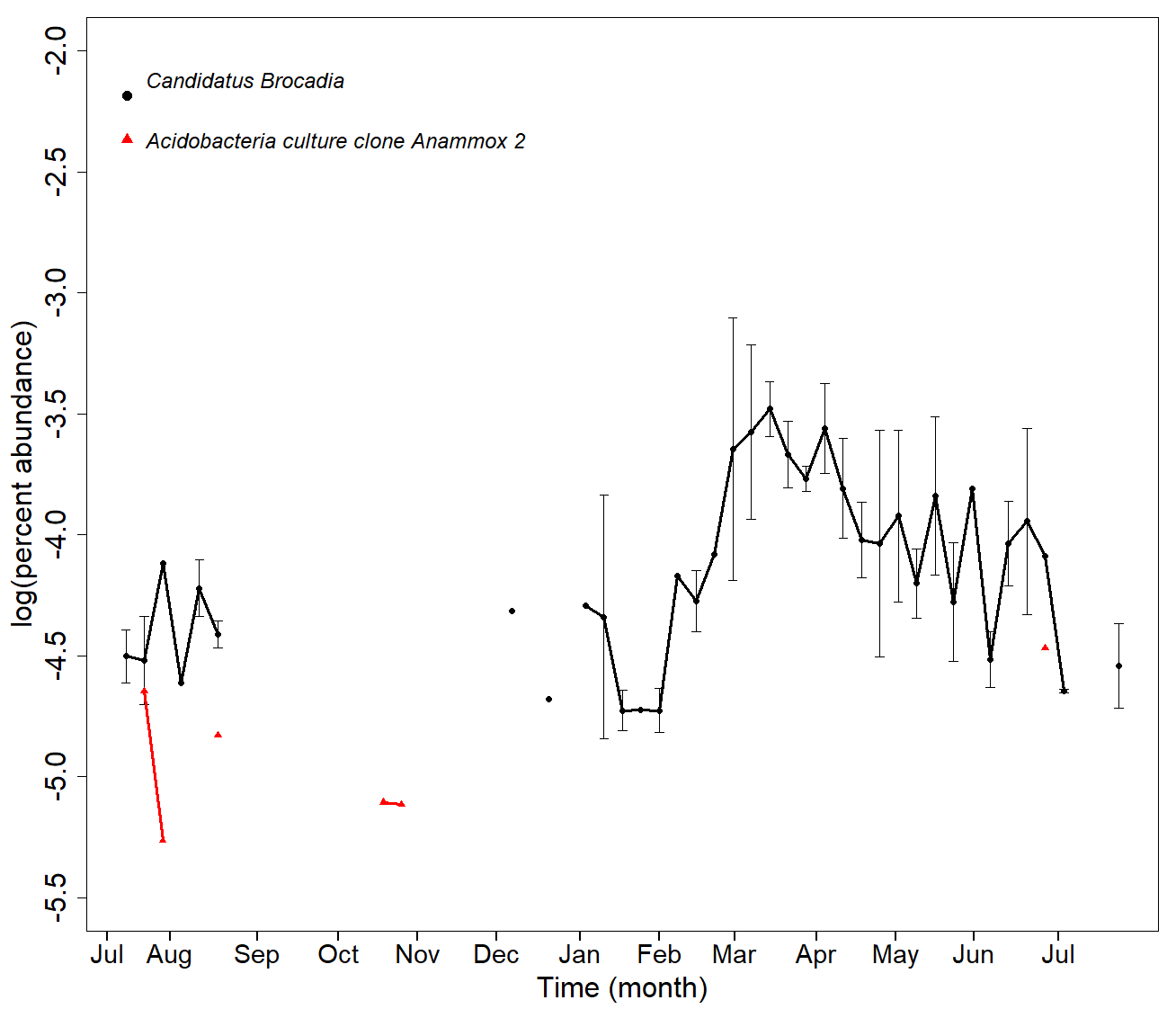


**Figure S7.** One OTU affiliated to a taxa comprising known anaerobic ammonia-oxidizing (anammox) bacterial was observed during the sample period, *Candidatus Broacadia* (black circles). A second OTU affiliated to an Acidobacteria clone from an Anammox enrichment is also included (red triangles). The relative sequence abundance of the *Candidatus Broacadia* OTU increases at the end of winter when nitrification performance decreased. During nitrification failure there is an abundance of both ammonia and nitrite in the reactors. The change in relative abundance of the *Candidatus Broacadia* OTU is probably not significant enough to be associated with nitrogen removal, however certain strains of *Candidatus Brocadia* thrive in colder temperatures (Hendrickx et al., 2014).


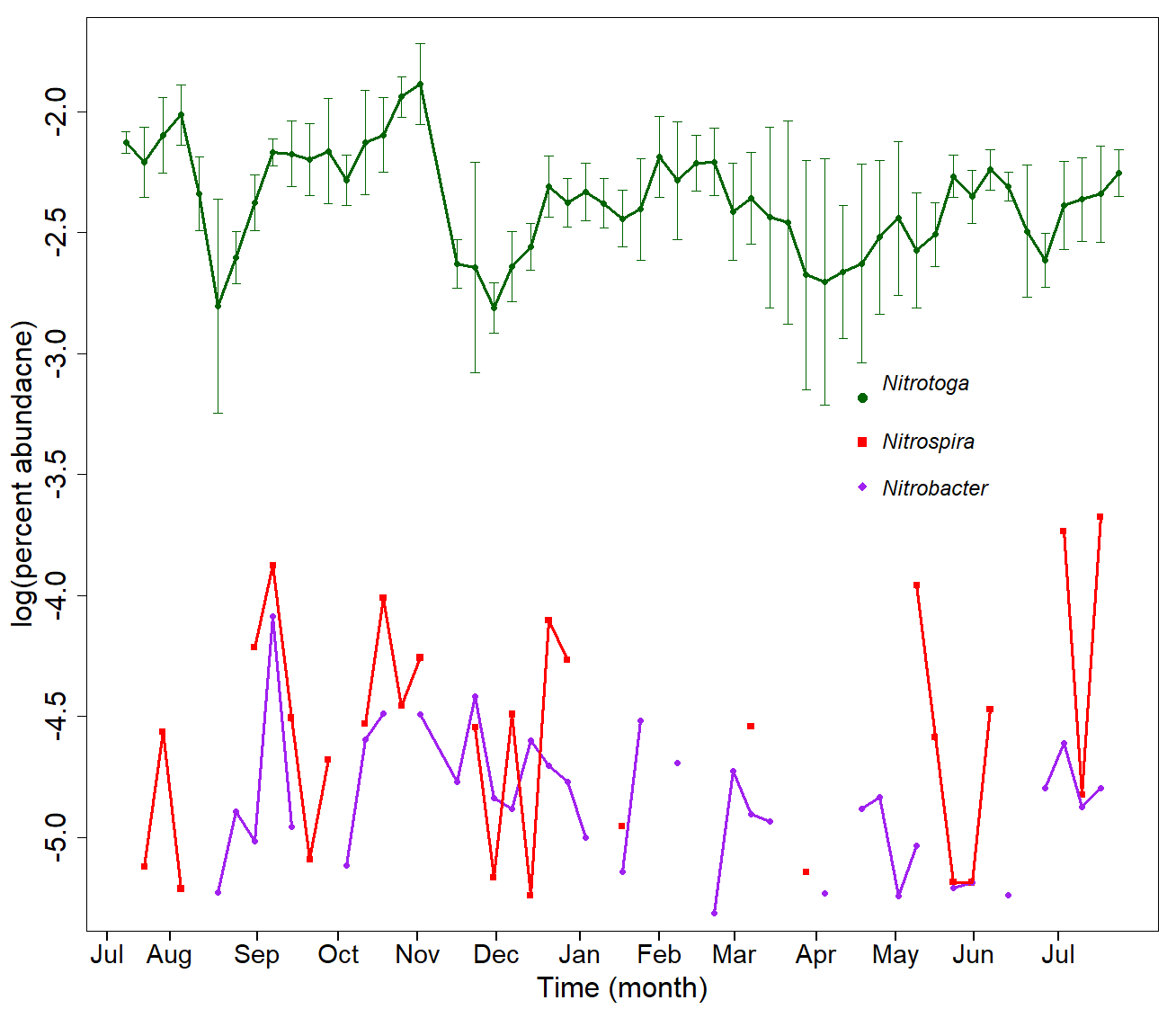


**Figure S8.** The known nitrite oxidizing community is highlighted over the months above. The dominance of *Nitrotoga* (green circles) over other nitrite oxidizing bacteria such as *Nitrospira* (red squares) and *Nitrobacter* (purple diamonds has been previously cultured from activated sludge and are ideal in temperatures between 10°C and 17°C (Alawi, Off, Kaya, & Spieck, 2009).


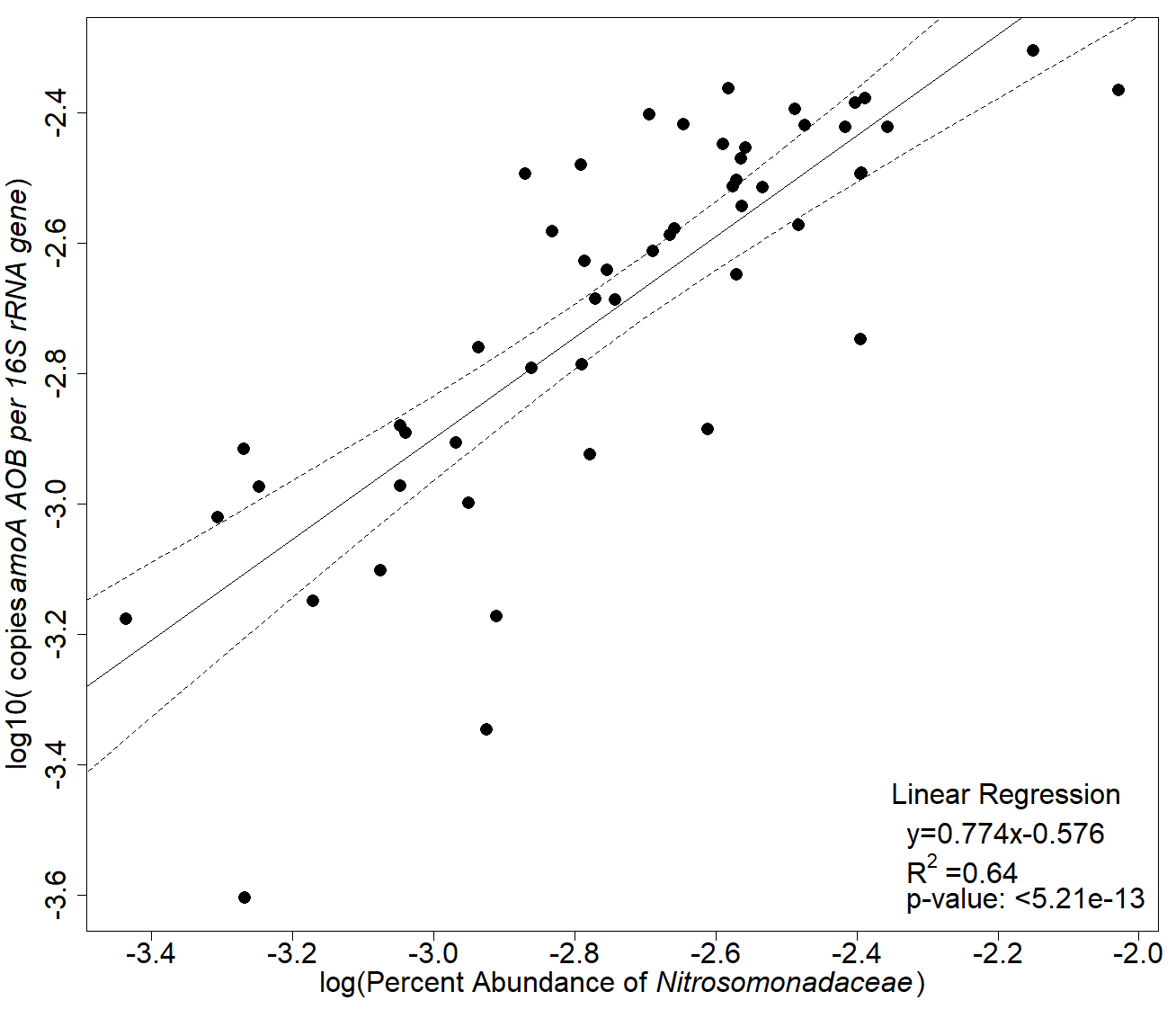


**Figure S9.** The percent abundance of *Nitrosomonadaceae* (x-axis) correlated to the copies of *amoA* (AOB) gene per *16S rRNA* gene (y-axis). The linear regression is shown in the solid line with the 95% confidence intervals denoted on the dashed line.

.
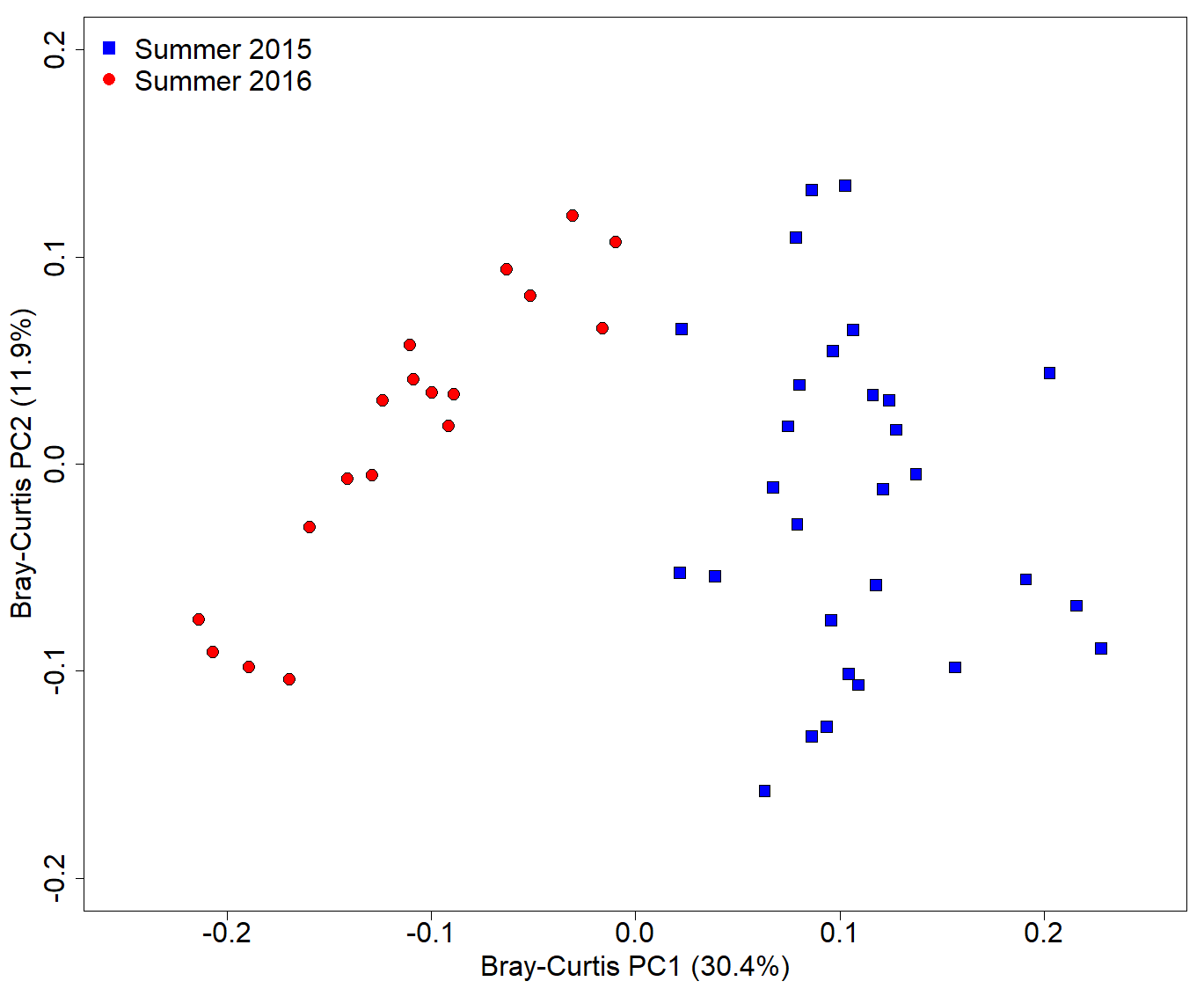


**Figure S10.** shows a principal coordinates analysis of only the two summers with July 2015 in blue, and July 2016 in red. The two separate and appear distinct, however they only overlap for three weeks. Summer 2015 started July 13^th^, 2015 until the end of summer. Summer 2016 started June 20^th^, 2016, and sampling finished July 30^th^ with only three overlapping weeks.


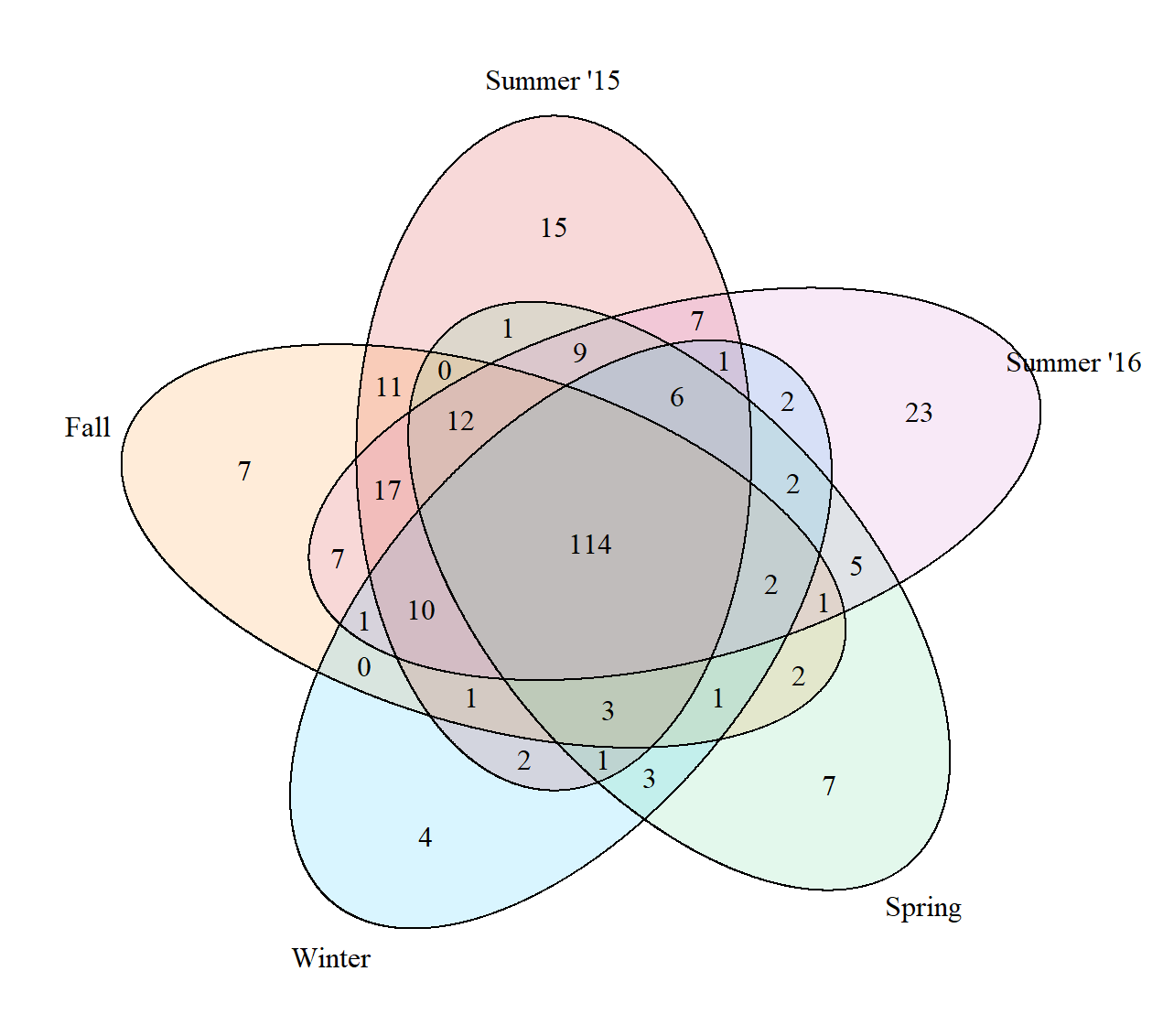


**Figure S11.** The quintuple venn diagram represents the core community, multiple season, and single season OTUs. Summer 2015 is represented in red, Fall in yellow, Winter in blue, Spring in green, and Summer 2016 in purple. The core 114 OTUs represent the shared space between all the seasons. Anything shared between two seasons is a multiple season OTU and listed only under each season is a single seasson OTU.

**Figure S12.** Bray-Curtis dissimilarity creates several dimensions where each dimension explains a portion of the total variance observed between samples. This plot highlights each individual dimension in the blue dots on the left y-axis. The orange dots are a cumulative total of the dimensions on the right y-axis.

**References:**

Alawi, M., Off, S., Kaya, M., & Spieck, E. (2009). Temperature influences the population structure of nitrite-oxidizing bacteria in activated sludge. *Environ Microbiol Rep, 1*(3), 184-190.

Gerda Harms, A. C. L., Heve M. Dionisi, Igrid R. Gregory, Victoria M. Garrett, Shawn A. Hawkins, Kevin G. Robinson, Gary S. Sayler. (2003). Real-Time PCR Quantification of Nitrifying Bacteria in a Municipal Wastewater Treatment Plant. *Environmental Science & Technology, 37*, 343-351.

Gesche Braker, A. F., Karl-Paul Witzel. (1998). Development of PCR Primer Systems for Amplification of Nitrite Reductase Genes (nirK and nirS) To Detect Denitrifying Bacteria in Environmental Samples. *Applied and Environmental Microbiology, 64*(10).

Harter, J., El-Hadidi, M., Huson, D. H., Kappler, A., & Behrens, S. (2017). Soil biochar amendment affects the diversity of nosZ transcripts: Implications for N2O formation. *Scientific Reports, 7*(1), 3338.

Hendrickx, T. L., Kampman, C., Zeeman, G., Temmink, H., Hu, Z., Kartal, B., & Buisman, C. J. (2014). High specific activity for anammox bacteria enriched from activated sludge at 10 degrees C. *Bioresour Technol, 163*, 214-221.

Meinhardt, K. A., Bertagnolli, A., Pannu, M. W., Strand, S. E., Brown, S. L., & Stahl, D. A. (2015). Evaluation of revised polymerase chain reaction primers for more inclusive quantification of ammonia-oxidizing archaea and bacteria. *Environ Microbiol Rep, 7*(2), 354-363.

Pjevac, P., Schauberger, C., Poghosyan, L., Herbold, C. W., van Kessel, M. A. H. J., Daebeler, A., . . . Daims, H. (2017). AmoA-Targeted Polymerase Chain Reaction Primers for the Specific Detection and Quantification of Comammox Nitrospira in the Environment. *Frontiers in Microbiology, 8*.

Throback, I. N., Enwall, K., Jarvis, A., & Hallin, S. (2004). Reassessing PCR primers targeting nirS, nirK and nosZ genes for community surveys of denitrifying bacteria with DGGE. *FEMS Microbiol Ecol, 49*(3), 401-417.

Ulrich Nubel, F. G.-P., Gerard Muyzer. (1997). PCR Primers To Amplify 16S rRNA Genes from Cyanobacteria. *Applied and Environmental Microbiology, 63*(8).
